# Dynamic transcriptome and DNA methylome analyses on *longissimus dorsi* to identify genes underlying intramuscular fat content in pigs

**DOI:** 10.1186/s12864-017-4201-9

**Published:** 2017-10-12

**Authors:** Yuding Wang, Cai Ma, Yi Sun, Yi Li, Li Kang, Yunliang Jiang

**Affiliations:** 1 Shandong Provincial Key Laboratory of Animal Biotechnology and Disease Control and Prevention, College of Animal Science and Veterinary Medicine, Shandong Agricultural University, No. 61 Daizong Street, Taian, 271018 People’s Republic of China; 2 Shandong Provincial Key Laboratory of Animal Disease Control and Breeding, Institute of Animal Science and Veterinary Medicine, Shandong Academy of Agricultural Sciences, Jinan, 250100 People’s Republic of China; 3 Central Hospital of Taian, Taian, 271018 People’s Republic of China

**Keywords:** Pig, *longissimus dorsi* muscle, Intramuscular fat content, Transcriptome, DNA methylome, Gene

## Abstract

**Background:**

The intramuscular fat content (IMF) refers to the amount of fat within muscles, including the sum of phospholipids mainly found in cell membranes, triglycerides and cholesterol, and is determined both by hyperplasia and hypertrophy of adipocyte during the development of pigs. The IMF content is an important economic trait that is genetically controlled by multiple genes. The Laiwu pig is an indigenous fatty pig breed distributed in North China, characterized by excessively higher level of IMF content (9%~12%), therefore, is suitable for the identification of genes controlling IMF variations. To identify genes underlying IMF deposition, we performed genome-wide transcriptome and methylome analyses on *longissimus dorsi* (LD) muscle in Laiwu pigs across four developmental stages.

**Results:**

A total of 22,524 expressed genes were detected and 1158 differentially expressed genes (DEGs) were hierarchically clustered in the LD muscle over four developmental stages from 60 d to 400 d. These genes were significantly clustered into four temporal expression profiles, and genes participating in fat cell differentiation and lipid biosynthesis processes were identified. From 120 d to 240 d, the period with the maximum IMF deposition rate, the lipid biosynthesis related genes (*FOSL1*, *FAM213B* and *G0S2*), transcription factors (TFs) (*EGR1*, *KLF5*, *SREBF2*, *TP53* and *TWIST1*) and enriched pathways (*steroid biosynthesis* and *fatty acid biosynthesis*) were revealed; and fat biosynthesis relevant genes showing differences in DNA methylation in gene body or intergenic region were detected, such as *FASN*, *PVALB*, *ID2*, *SH3PXD2B* and *EGR1*.

**Conclusions:**

This study provides a comprehensive landscape of transcriptome of the LD muscle in Laiwu pigs ranging from 60 to 400 days old, and methylome of the LD muscle in 120 d and 240 d Laiwu pigs. A set of candidate genes and TFs involved in fat biosynthesis process were identified, which were probably responsible for IMF deposition. The results from this study would provide a reference for the identification of genes controlling IMF variation, and for exploring molecular mechanisms underlying IMF deposition in pigs.

**Electronic supplementary material:**

The online version of this article (10.1186/s12864-017-4201-9) contains supplementary material, which is available to authorized users.

## Background

Pigs are the main sources for human meat consumption and provide 43% of total meat production in the world [1, 2]. Skeletal muscle is the primary meat production tissue of pigs, responsible for 20%~50% of their body mass [3]. The intramuscular fat content (IMF) refers to the amount of fat within muscles, which includes the sum of phospholipids mainly found in cell membranes, triglycerides serving as the main forms of energy reserves and cholesterol [4]. The triglycerides in mammalian and avian muscles are mainly stored within intramuscular adipocytes and myofibres cytoplasm in droplets in close vicinity to mitochondria [4–6], which appear as the flecks and streaks of fat within the lean sections of meat [4, 7]. The IMF is considered as late developing fat storage, which is determined both by hyperplasia and hypertrophy of adipocyte during the development of pigs [8]. The fatty acid composition of these fat components in muscle tissue is associated with eating quality [9]; saturated and monounsaturated fatty acids (SFA and MUFA) are positively, while polyunsaturated fatty acids (PUFA) are negatively correlated with meat flavour [10].

The IMF content is a polygenic trait in livestock species that affects meat quality traits such as flavour, drip loss and shear force [4, 11]. Studies on the genetic basis of IMF in pigs have been extensively carried out, and some genes associated with IMF were reported [12, 13], however, the molecular mechanisms of porcine IMF are still unclear. The Laiwu pig is an indigenous fatty pig breed distributed in North China, characterized by excessively higher level of IMF content (9%~12%), far exceeding the commercial lean pigs [14], therefore, is suitable for the identification of genes controlling IMF variations. Several studies have analyzed the genetic basis of IMF deposition in Laiwu pigs using candidate gene approach [15–17]. Recently, genome-wide analysis of differentially expressed genes (DEGs) has been proved as an effective approach to elucidating genetic mechanisms of complex traits. The transcriptome of porcine subcutaneous adipose [18, 19], muscle [2, 20] and liver [21, 22] have been investigated, while genome-wide analysis on the molecular mechanisms of IMF deposition in Laiwu pigs is still lacking.

In this study, we present a comprehensive survey of the transcriptome profiles of the *longissimus dorsi* muscle (LD) in Laiwu pigs across 60, 120, 240 and 400 days of age, which are representative of major morphological and developmental stages in Laiwu pigs. Moreover, DNA methylation at CpG dinucleotide in the promoter region plays an important role in regulating the transcription of genes [21, 23]. Based on the transcriptome results, we further compared the changes in DNA methylation profile during the fastest fat deposition period (from 120 d to 240 d) and analyzed the role of DNA methylation in the expression dynamics of genes related to IMF changes. The results of this study revealed several genes and pathways involved in adipogenesis and lipogenesis in LD muscle that likely underlie IMF trait in pigs.

## Results

### Dynamics in IMF and fatty acid composition of porcine LD muscle across four developmental stages

**Fig. 1:**
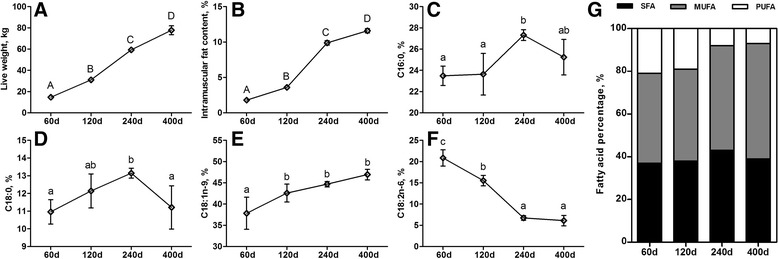
The phenotypic characteristics in the *longissimus dorsi* (LD) muscle of Laiwu pigs. Dynamics in the (**a**) live weight, (**b**) intramuscular fat (IMF) content and fatty acid composition of (**c**) palmitic acid (C16: 0), (**d**) stearic acid (C18: 0), (**e**) oleic acid (C18: 1n-9) and (**f**) linoleic acid (C18: 2n-6) across 60, 120, 240 and 400 days of age. (**g**) The proportion of saturated fatty acids (SFA), monounsaturated fatty acids (MUFA) and polyunsaturated fatty acids (PUFA). The data show the means ± SD analyzed by one-way ANOVA followed by Duncan’s multiple comparison. a-c: *p* ≤ 0.05, A-D: *p* ≤ 0.01

From 60 d to 400 d, along with the growth of pigs, the IMF content of porcine LD muscle increased significantly (*p* < 0.01), and from 120 d to 240 d, it is increased from 3.59% to 9.88%, representing the fastest fat deposition stage of the LD muscle in Laiwu pigs (Fig. 1a, b). The saturated fatty acid proportion of both palmitic acid (C16: 0) and stearic acid (C18: 0) significantly increased from 120 d to 240 d, then decreased at 400 d (Fig. 1c, d). Oleic acid (C18: 1) is the most abundant unsaturated fatty acid, accounting for approximately 40% of total muscle fatty acids and increased slightly over time (Fig. 1e). By contrast, the proportion of linoleic acid (C18: 2n-6) decreased significantly from 60 d to 240 d (Fig. 1f). The SFA and MUFA accounted for relatively larger proportion of fatty acid in LD muscle from 240 d to 400 d, whereas the opposite is for the PUFA (Fig. 1g).

### Summary of RNA-seq sequencing

**Fig. 2:**
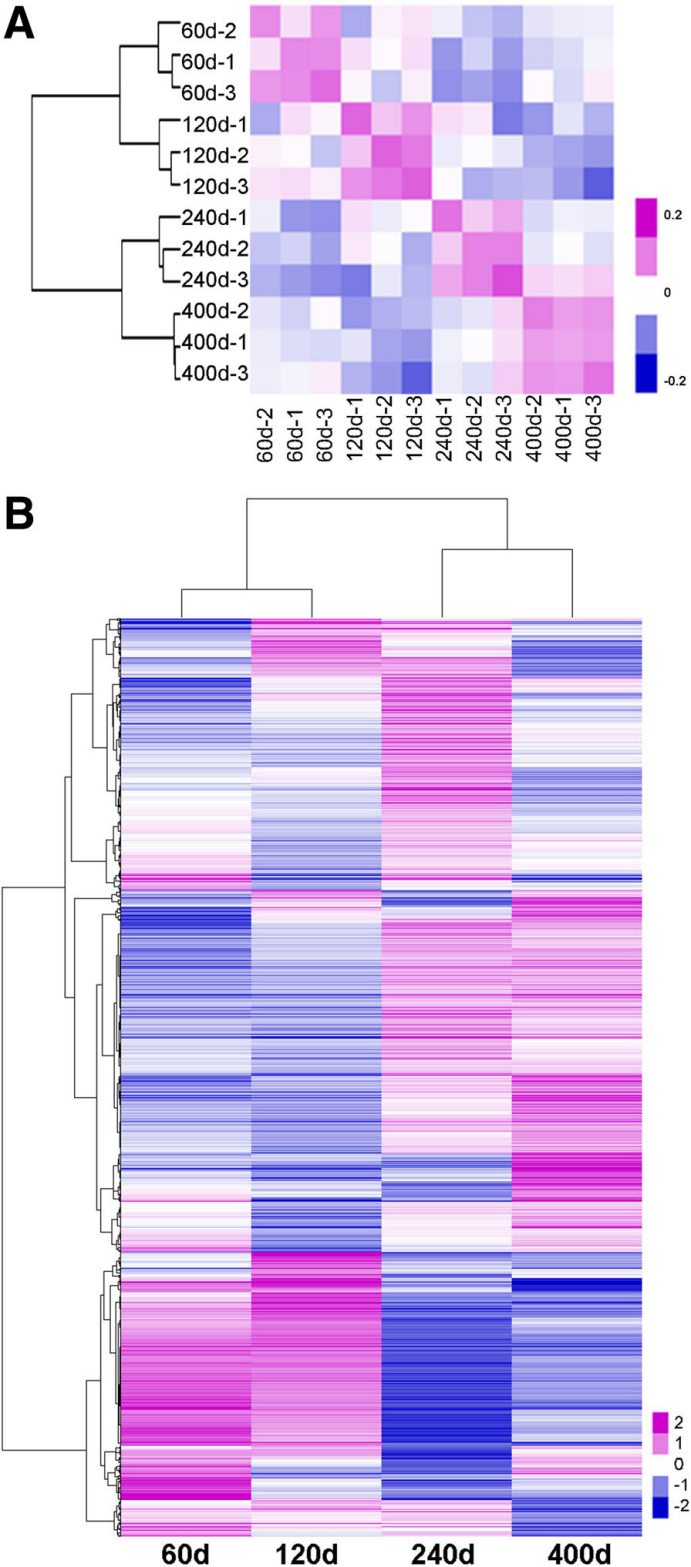
Hierarchical clustering analysis for all the samples and differentially expressed genes (DEGs). **a** Heat map matrix of 12 LD muscle samples of Laiwu pigs constructed using Pearson’s correlation. **b** Hierarchical clustering analysis for DEGs in the *longissimus dorsi* (LD) muscle of Laiwu pigs across four developmental stages from 60 d to 400 d

High-throughput RNA-seq generated 54.19 Gb clean data for 12 LD muscle samples of Laiwu pigs across four postnatal developmental stages of 60, 120, 240 and 400 d, and 70.15%–73.61% reads were successfully aligned to pig genome. All 12 samples had at least 88.47% reads equal to or exceeding Q30 (Table 1). The pair wise correlation was more than 0.83 among individuals of each group (Fig. 2a). A total of 22,524 expressed genes were detected and 1158 DEGs were hierarchically clustered over four developmental stages (Fold-Change ≥ 2 and FDR ≤ 0.01) (Fig. 2b). Six genes including *C/EBPA*, *C/EBPD*, *MSTN*, *PDK4*, *PPARG* and *SCD* were chosen and quantified using qRT-PCR to validate sequencing data, and a strong correlation (*r* > 0.76) between qRT-PCR and RNA-seq data was observed, suggesting that the RNA-seq result was reliable (Additional file 1: Figure S1).

**Table 1:** Summary of RNA-Seq metrics from transcriptomes across four developmental stages of Laiwu pigs

Sample	Total reads	Mapped reads	Mapped ratio, %	Uniq mapped reads	Uniq mapped ratio, %	GC content, %	% ≥ Q30
60 d-1	33,532,956	23,886,799	71.23	22,600,471	67.40	54.87	88.37
60 d-2	39,230,000	28,092,483	71.61	26,588,906	67.78	54.53	88.43
60 d-3	36,445,032	26,013,238	71.38	24,488,820	67.19	55.48	89.10
120 d-1	34,665,986	24,908,793	71.85	23,410,399	67.53	55.01	88.67
120 d-2	34,780,852	24,399,466	70.15	22,902,426	65.85	55.43	88.48
120 d-3	33,425,342	23,754,838	71.07	22,465,927	67.21	55.37	88.53
240 d-1	33,403,006	23,859,687	71.43	22,383,982	67.01	53.83	88.66
240 d-2	34,412,034	25,331,129	73.61	23,840,969	69.28	53.35	89.17
240 d-3	34,893,280	25,348,989	72.65	23,881,912	68.44	53.08	89.18
400 d-1	42,825,118	30,631,112	71.53	28,834,821	67.33	53.24	89.21
400 d-2	36,291,654	25,749,722	70.95	24,213,599	66.72	52.91	88.47
400 d-3	36,313,474	26,147,929	72.01	24,557,825	67.63	53.48	89.06

### Classification of the expressed genes in porcine LD muscle

**Fig. 3:**
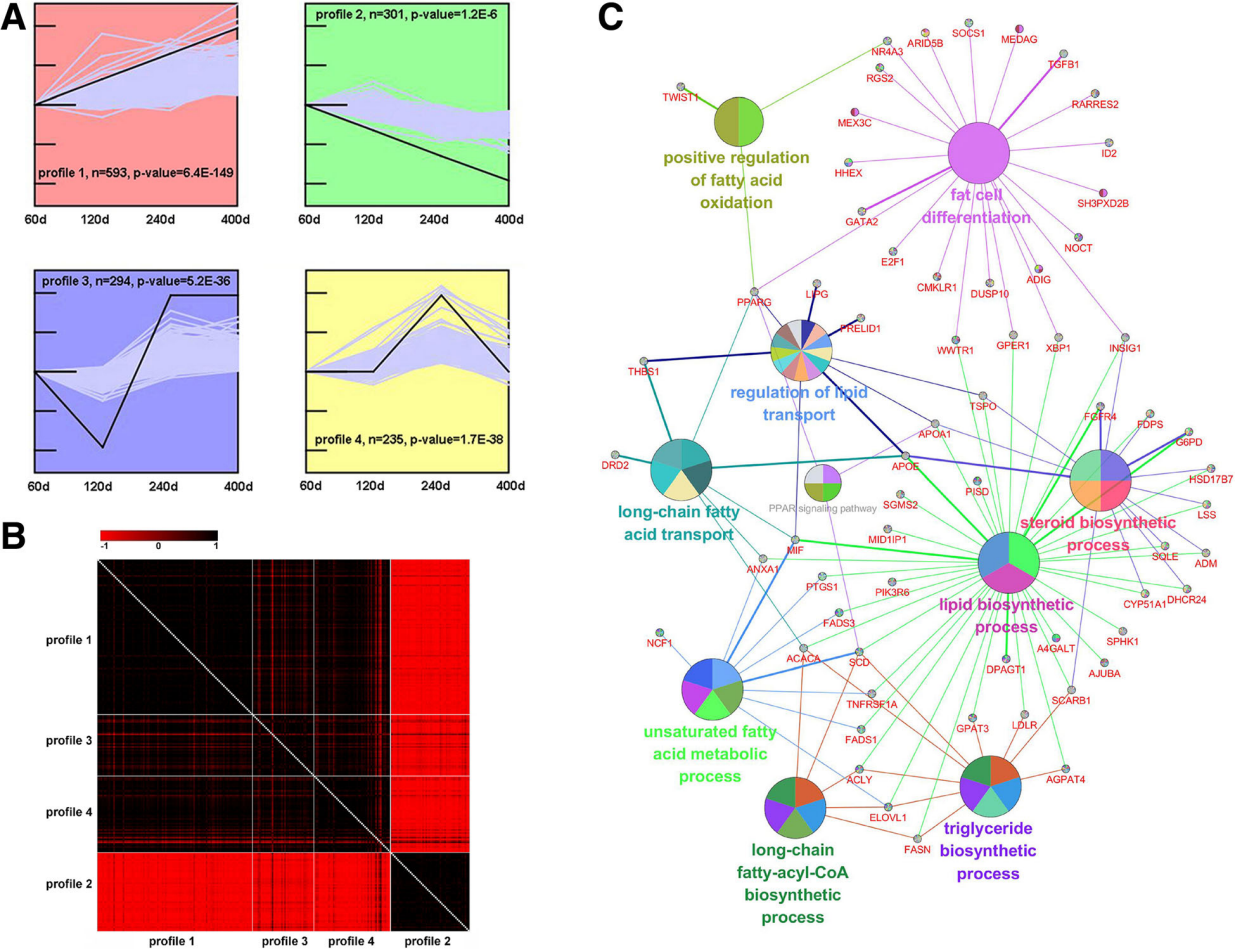
Analysis of short time-series gene expression cluster across four developmental stages. **a** Four significant cluster profiles under the order of time points. ‘*n*’ represents the gene numbers of each profile. **b** The relationship between these four significant clustered profiles was calculated based on correlation coefficients. Strongly positive or negative correlations between gene pairs are shown in black or red, respectively. **c** The differentially expressed genes and functions associated with fat metabolism in profile 1, 3 and 4. GO terms and genes are represented as nodes based on their kappa score more than 0.4 and networks with at least three nodes. The node size represents the GO terms enrichment significance

We clustered the expressed genes (at least one group FPKM ≥ 0.1) in the LD muscle of Laiwu pigs using Short Time-series Expression Miner (STEM) to obtain statistically significant clustered temporal expression profiles [24]. A total of 10 expression profiles were obtained, and the first four profiles were significantly clustered, including 1423 genes (*p* < 0.01, Fig. 3a). Among these profiles, profile 1, 3 and 4 showed a robust correlation and an expression pattern similar to IMF variations in the LD muscle of Laiwu pigs from 60 d to 400 d (Fig. 3b). The genes from these three files (Additional file 2: Table S1) were subsequently uploaded into ClueGO, and those genes participating in fat cell differentiation, lipid metabolism processes and skeletal muscle development were identified, including *ACACA*, *SCD*, *ACLY*, *ELOVL1* and *FASN* that participate in long-chain fatty-acyl-CoA, triglyceride and lipid biosynthetic processes; *APOE*, *APOA1*, *TSPO*, *FGFR4*, *FDPS*, *G6PD*, *HSD17B7*, *LSS*, *ADM*, *SQLE*, *DHCR24*, *CYP51A1* and *SCARB1* that are involved in steroid biosynthetic processes (Fig. 3c); *NFATC2*, *TMEM8C*, *MYOG*, *MYOD1*, *MYF5* and *MYF6* that are involved in myoblast fusion and *ID2*, *TNNT2*, *MEGF10* and *MYL3* that participate in muscle tissue development (Additional file 3: Figure S2).

### Expression analysis on IMF relevant QTL in the LD muscle of Laiwu pigs

To validate the DEGs potentially related to pig IMF content trait, we examined the expression changes of IMF content relevant QTL across four development stages of Laiwu pigs based on pig QTL database (http://www.animalgenome.org/cgi-bin/ QTLdb/SS/index). A total of 14 specifically expressed genes in LD muscle were detected, most of them have the highest FPKM value at 240 d, including *SCD*, *FASN*, *FADS2*, *ANK1*, *ACSF3* and *SNAI2*, and three genes including *PIK3R6*, *PIK3C3* and *CA3* showed the peak FPKM value at 400 d (Table 2).

**Table 2:** The gene expression changes of IMF relevant QTLs across four developmental stages of Laiwu pigs

Chr.^1^	Gene	log_2_(FC)^2^		
		60 d vs 120 d	120 d vs 240 d	240 d vs. 400 d
X	*SLC9A7*	−1.44**	0.57	0.29
14	*SCD*	0.28	1.65***	−1.26***
12	*PIK3R6*	0.27	0.15	1.42**
6	*PIK3C3*	0.12	−1.15***	0.65*
3	*LCLAT1*	−0.79**	0.38	−0.68*
12	*FASN*	1.72***	1.79***	−1.75***
2	*FADS2*	−0.16	1.02**	−0.24
6	*FABP3*	0.80	−0.96	2.48
5	*ATP5B*	0.12	0.45	−0.84**
17	*ANK1*	−0.40	0.91**	0.02
6	*ACSF3*	−0.13	0.90**	−0.58*
4	*SNAI2*	0.24	1.37*	0.15
4	*CA3*	−0.35	0.10	0.82*
12	*MYH3*	0.86	0.94	−0.61

^1^Chr: Chromosome of gene located

^2^FC: FPKM fold-change between different groups. *p < 0.05; **p < 0.01; ***p < 0.001

### DEGs and the pathway enrichment between adjacent developmental stages in porcine LD muscle

**Fig. 4:**
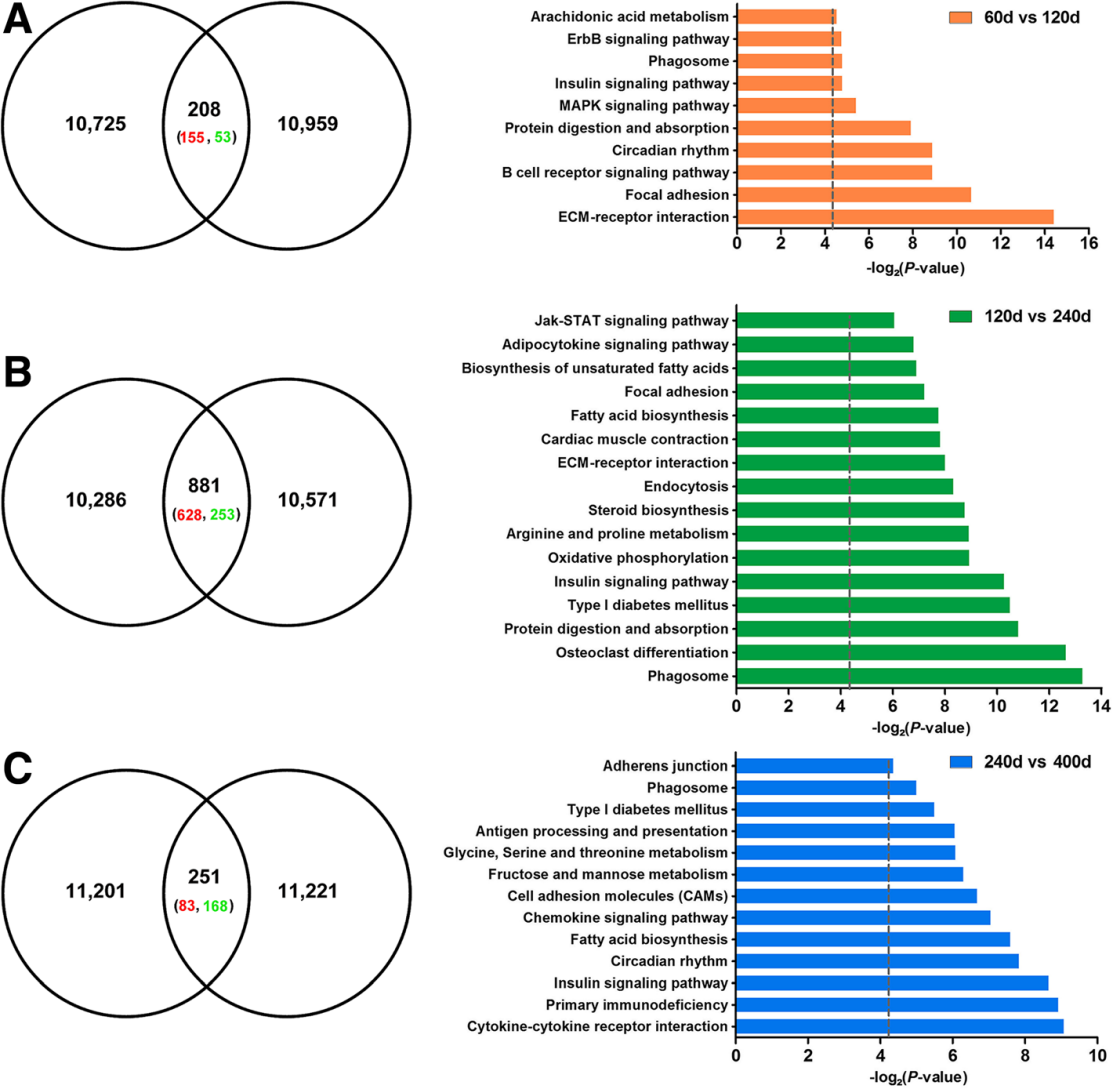
The differentially expressed genes and enriched pathways in the LD muscle of Laiwu pigs. **a** 60 d vs 120 d, (**b**) 120 d vs 240 d and (**c**) 240 d vs 400 d. The number in circle represent the detected expressed gene number, the intersection was the differentially expressed gene number, red and green represented the up-regulated and down-regulated genes, respectively. Grey dashed line in pathway indicates a threshold of *p* = 0.05

The DEGs between adjacent developmental stages, i.e., 60 d vs 120 d, 120 d vs 240 d and 240 d vs 400 d, were screened (Additional file 4: Table S2), and pathways with these DEGs were performed using DAVID. From 60 d to 120 d, 208 genes were differentially expressed, including 155 up-regulated and 53 down-regulated genes, which are significantly enriched in 11 pathways including cell proliferation (*ECM-receptor interaction*, *focal adhesion*) and immune (*B cell receptor signalling pathway*) (*p* < 0.01) (Fig. 4a). From 120 d to 240 d, 881 genes were differentially expressed, including 628 up-regulated and 253 down-regulated genes, which are involved in 16 pathways including phagocytosis (*phagosome*, *endocytosis*), bone differentiation (*osteoclast differentiation*), energy metabolism and fat deposition (*type I diabetes mellitus*, *insulin signalling pathway*, *steroid biosynthesis*, *fatty acid biosynthesis*) and protein metabolism (*protein digestion and absorption*, *arginine and proline metabolism*) (*p* < 0.01) (Fig. 4b). From 240 d to 400 d, 251 genes were differentially expressed, including 83 up-regulated and 168 down-regulated genes, which are involved in 13 pathways including immune (*cytokine-cytokine receptor interaction*, *primary immunodeficiency*, *chemokine signalling pathway*), energy metabolism and fat deposition (*insulin signalling pathway*, *fatty acid biosynthesis*) (*p* < 0.01) (Fig. 4c). Pathways related to fat deposition, including *fatty acid biosynthesis*, *steroid biosynthesis*, *biosynthesis of unsaturated fatty acid* and *adipocytokine signalling pathways*, were revealed in the comparison between 120 d and 240 d. The *fatty acid biosynthesis* pathway was also significantly enriched in 400 d, but most of the DEGs were down-regulated.

### Functional enrichment in porcine LD muscle between 120 d and 240 d

**Fig. 5:**
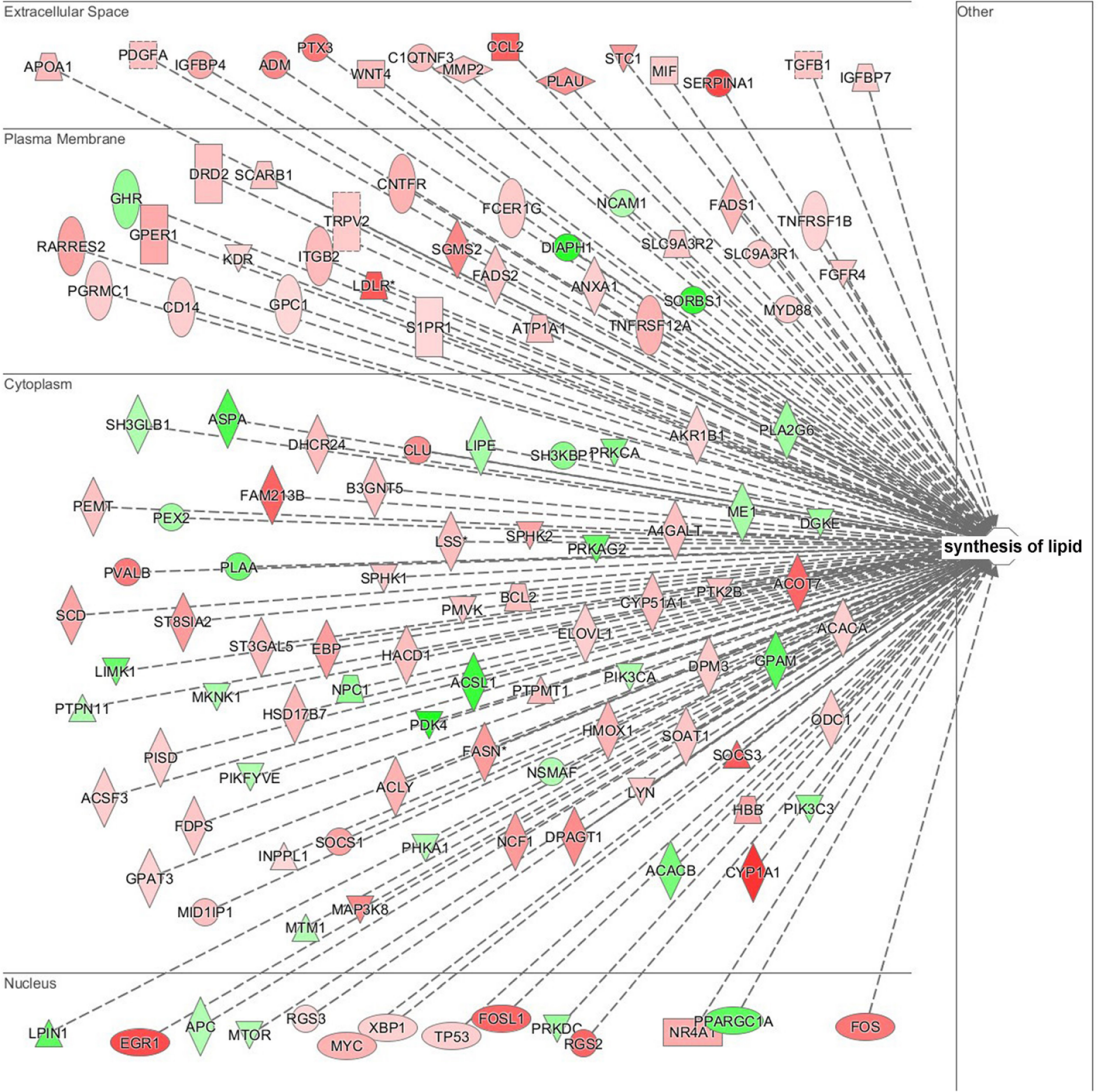
Candidate genes associated with fat biosynthesis process between 120 d and 240 d. The DEGs are organized by cellular substructure, up-regulated genes in 240 d are indicated in red, down-regulated genes in 240 d are indicated in green and the colour depth represents the regulated degree. IPA predicted activation Z-score = 1.25, *p*-value = 3.73E-10

From 120 d to 240 d, the fastest IMF deposition stage, the top 10 significantly up- or down-regulated DEGs were found to be involved in biological process such as energy metabolism (*ATP5J2*, *DNAJB1*), transcription (*EGR1*, *KLF11*), muscle development (*CHRNG*, *TNNT2*) and fat metabolism (*G0S2*, *CYP1A1*, *FAM213B*) (Table 3). We uploaded 881 DEGs to IPA for functional analysis and revealed 127 DGEs (93 up-regulated and 34 down-regulated) that are involved in lipid synthesis process (Z-score = 1.25, *p* < 0.001). Among those genes related to lipid synthesis, the top 5 up-regulated genes were *CYP1A1*, *SERPINA1*, *LDLR*, *EGR1* and *FOSL1*, and the top 5 down-regulated genes were *DIAPH1*, *SORBS1*, *PDK4*, *ACSL1* and *ASPA* (Fig. 5), and 84 genes were included in profiles 1, 3 and 4 (Additional file 5: Table S3), such as *FASN*, *ACACA*, *ACLY* and *SCD.*

**Table 3:** The top 10 up- and down-regulated genes of 120 d vs 240 d Laiwu pigs

Regulate	Gene	FPKM (120 d)	FPKM (240 d)	Log_2_(FC)^a^	Function
Up	CHRNG	0.23	7.45	5.04	GO:0006936: muscle contraction
	CYP1A1	0.15	2.43	4.07	GO:0008395: steroid hydroxylase activity
	ISG15	4.48	65.3	3.86	KEGG Pathway: RIG-like receptor signalling pathway
	DNAJB1	14.66	197.69	3.75	GO:0001671: ATPase activator activity
	NREP	21.21	240.52	3.5	GO:0017015: Regulation of transforming growth factor beta receptor signaling pathway
	EGR1	11.7	111.05	3.25	GO:0006366: Transcription from RNA polymerase II promoter
	G0S2	5.16	47.34	3.2	Reactome Pathway: Regulation of lipid metabolism by Peroxisome proliferator-activated receptor alpha
	TNNT2	8.55	70.02	3.03	Reactome Pathway: Striated muscle contraction
	ATP5J2	1	8.01	3.02	GO:0006754: ATP biosynthetic process
	FAM213B	87.24	616.05	2.82	KEGG Pathway: Arachidonic acid metabolism
Down	A2M	11.94	0.18	−6.08	GO:0004867: serine-type endopeptidase inhibitor activity
	CDH2	38.08	3.01	−3.66	GO:0008013: beta-catenin binding
	NOS1	7.36	0.7	−3.4	GO:0006809: nitric oxide biosynthetic process
	ZIC3	1.32	0.13	−3.35	GO:0007368: determination of left/right symmetry
	PDK4	722.31	96.27	−2.91	GO:0004672: protein kinase activity
	FBXO32	53.3	7.7	−2.79	KEGG Pathway: FoxO signalling pathway
	KLF11	7.56	1.18	−2.68	GO:0044212: transcription regulatory region DNA binding
	DIAPH1	89.73	18.76	−2.26	GO:0005815: microtubule organizing centre
	UCP3	111.83	23.33	−2.26	GO:0017077: oxidative phosphorylation uncoupler activity
	SORBS1	73.38	16.07	−2.19	KEGG Pathway: PPAR signalling pathway

^a^FC: Fold-Change of FPKM in transcriptome data in 120 d vs 240 d

Furthermore, the upstream key regulators of IMF deposition were predicted using IPA [2, 8], considering gene expression is transcriptionally controlled by upstream regulators, particularly transcription factors (TFs). Even if TFs do not always exhibit some extent change, the expression of downstream genes would vary significantly [8]. A total of 1620 upstream regulators were predicted and 16 regulators would be expected to have a greater impact on IMF deposition based on their biological function annotations (Table 4). Meanwhile, the predicted downstream genes (Additional file 6: Table S4) and the binding site (Additional file 7: Table S5) of TFs were summarized. The TFs including *EGR1*, *KLF5*, *SREBF2*, *TP53* and *TWIST1* were activated (Z-score ≥ 2.0) [25], and the expression of *EGR1* and *TP53* was significantly up-regulated in 240 d compared with 120 d.

**Table 4:** Upstream regulators in the LD muscle of Laiwu pigs from 120 d to 240 d

Upstream regulator	Molecule type	Transcription		IPA prediction	
		Log_2_(FC)^a^	*p*-vale	Z-score^b^	*p*-value
*CYR61*	other	1.94	5.00E-05	1.82	1.17E-06
*DRD2*	G-protein coupled receptor	1.10	7.15E-03	0.51	3.96E-02
*EGR1*	transcription regulator	3.25	5.00E-05	3.39	1.23E-11
*FGF1*	growth factor	−0.85	1.08E-02	2.25	5.02E-05
*GPER1*	G-protein coupled receptor	1.54	3.00E-04	2.53	1.46E-04
*ID2*	transcription regulator	1.27	1.50E-04	−1.15	1.98E-04
*KLF5*	transcription regulator	0.66	1.00E-01	2.72	6.63E-05
*KLF6*	transcription regulator	0.25	3.36E-01	1.43	4.23E-04
*LDLR*	transporter	3.00	5.00E-05	1.00	1.66E-03
*MIF*	cytokine	0.93	2.00E-04	0.88	4.98E-02
*MLXIPL*	transcription regulator	−0.04	9.10E-01	1.97	3.64E-05
*PLIN5*	other	−0.16	5.72E-01	2.22	4.55E-02
*POMC*	other	0.58	9.77E-02	1.86	2.59E-03
*SREBF1*	transcription regulator	0.25	3.71E-01	1.45	5.53E-09
*SREBF2*	transcription regulator	1.13	3.99E-01	3.51	8.43E-05
*TP53*	transcription regulator	0.85	3.05E-03	2.67	4.83E-42
*TWIST1*	transcription regulator	0.64	3.13E-01	3.26	1.06E-11

^a^FC: Fold-Change of FPKM in transcriptome data between two groups

^b^Z-score: An IPA score method that reflects the activation state of predicted regulators

### mRNA comparison of Laiwu pigs with other pig breeds at 120 d

**Fig. 6:**
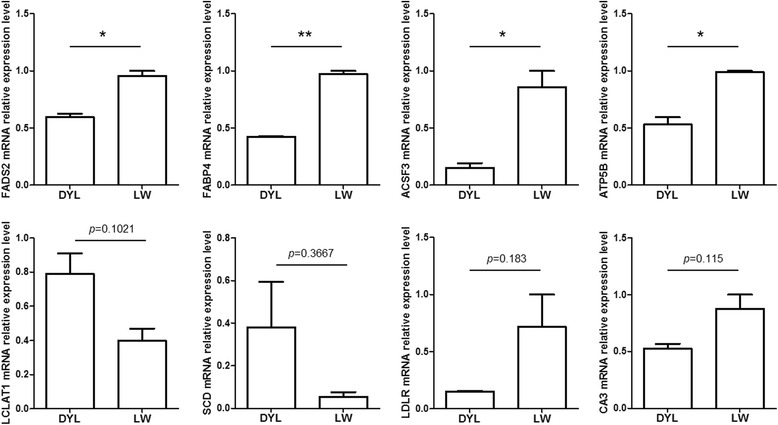
The detection of IMF candidate genes between DYL and Laiwu pigs using qRT-PCR. *p < 0.05; **p < 0.01

At 120 d, IMF content in the LD muscle of Laiwu pigs was significantly higher than that of DYL pigs (Additional file 8: Figure S3). The qRT-PCR analysis showed that the mRNA expression of *FADS2*, *FABP4*, *ACSF3* and *ATP5B* in LD muscle was significantly higher in Laiwu pigs than in DYL pigs, while that of *LCLAT1*, *SCD*, *LDLR* and *CA3* was not significantly different (Fig. 6). Meanwhile, Ovilo [26] has reported that the IMF of IB × DU pig was 2.87% at 120 d, which was lower than that of Laiwu pig (3.59%) at the same age. The transcriptome data of IB × DU pig at 120 d were downloaded from GEO of NCBI (GSE86441). A total of 682 DEGs were screened (Additional file 9: Table S6), and 49 IMF candidate genes were obtained based on their GO or KEGG annotations, of which 37 genes were higher and 11 genes were lower in the LD muscle of Laiwu pigs compared with IB × DU pigs (Additional file 10: Table S7).

### Landscape of DNA methylomes of porcine LD muscle

**Fig. 7:**
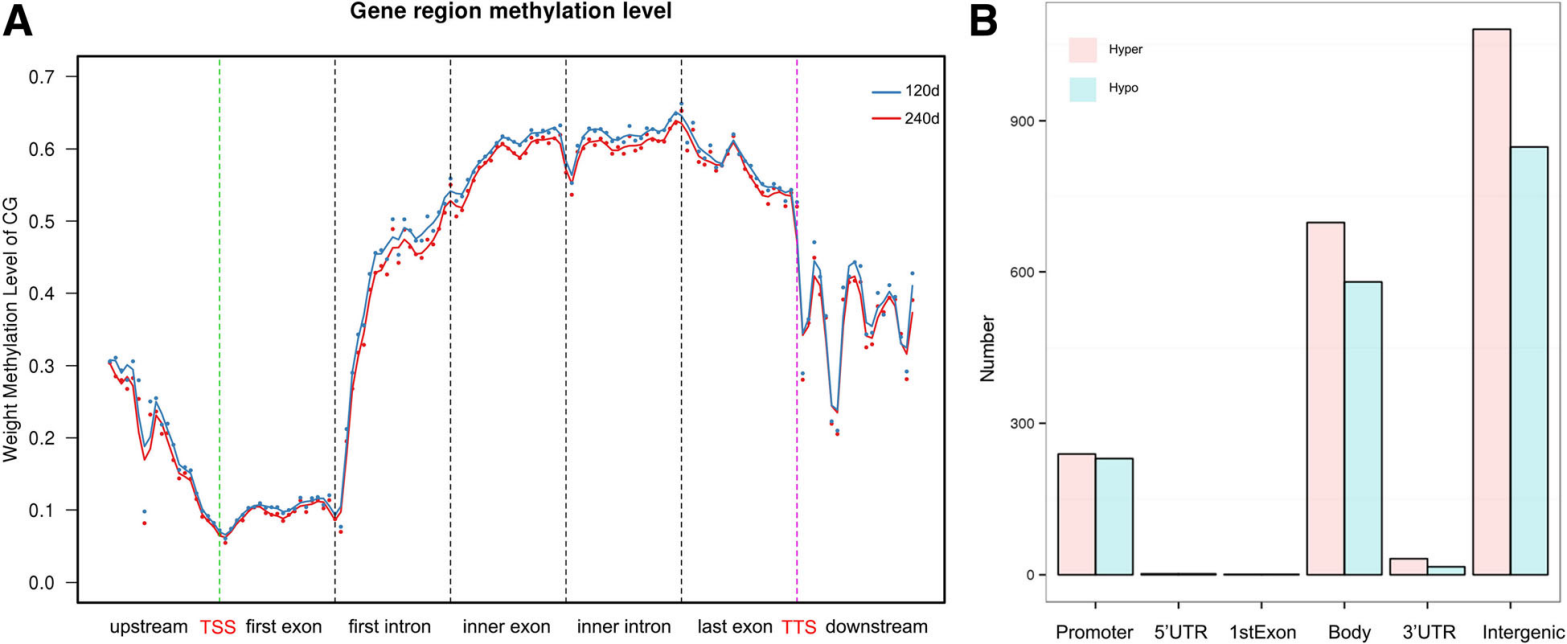
The methylation distribution around gene body of Laiwu pigs between 120 d and 240 d. **a** Methylation level of different sites around gene body. **b** Distribution of differentially methylated regions (DMRs) in gene body and intergenic region. Most (52.5%) DMRs located in intergenic region, followed by gene body (34.7%) and promoter (24.3%) regions. TSS: transcription start sites; TTS: transcription termination site

To further understand the role of DNA methylation in regulating the transcription of genes related to IMF deposition from 120 d to 240 d, we performed reduced representation bisulfite sequencing (RRBS) on the genomic DNA from the LD muscle of Laiwu pigs. Approximately 12.2G clean data were obtained in each group with 46.27% to 46.64% clean reads uniquely mapped (Table 5). We plotted the genome-wide distribution of cytosine according to sequencing read coverage and depth across chromosomes (Additional file 11: Figure S4) and the methylation distribution of genes within different functional components, including 2 kb region upstream, gene body and downstream region (Fig. 7a). Similar CpG methylation trend was shown between 120 d and 240 d: a relative low methylation levels were found in upstream region, reaching the lowest level around transcription start sites (TSS), being stable in the first exon, followed by a sharp increase in the first intron, and hitting a plateau till transcription termination site (TTS).

**Table 5:** Data generated by RRBS of LD muscle in 120 d and 240 d Laiwu pigs

Sample	Clean reads	Clean base	GC (%)	Q30 (%)	Unique mapped	Mapped (%)	Conversion rate (%)
120 d	44,613,858	12,393,351,502	31.90	85.43	20,643,510	46.27	99.49
240 d	43,556,079	12,047,950,580	32.71	85.12	20,316,081	46.64	99.50

A total of 124,693 differentially methylated cytosines (DMCs) between 120 d and 240 d were identified, with sequencing depth ≥ 4, and FDR ≤ 0.05 (Additional file 12: Table S8). Similarly, a total of 3676 differentially methylation regions (DMRs) were generated corresponding to 2303 genes. The DMRs were screened by the sequencing depth ≥ 10×, at least 3 DMCs, with the minimum value ≥0.3, and *p* < 0.05 using Fisher’s exact test. Then, these DMRs were annotated according to the up- or down-stream 3000 bp of gene using MOABS method [27] (Additional file 13: Table S9). Distribution of the DMRs showed that intergenic region accounted for the most (52.5%) methylated variations, followed by gene body (34.7%), and gene promoter (24.3%) (Fig. 7b). The number of DMRs distributed on genes was shown in Table 6, hyper-methylated regions generally exceeding hypo-methylated regions in 240 d than 120 d. In addition, we performed a GO functional enrichment analysis for genes with DMRs in their promoters [28]. The top 12 significantly enriched GO terms were mainly involved in transcription, myotube differentiation and osteoblast differentiation (Table 7). Notably, the *fat metabolism process* was also significantly enriched, the involved genes include *FASN*, *LEP*, *LCN12*, *GDPD3* and *FADS6*.

**Table 6:** Number of differentially methylated genes in DMRs in the LD muscle of Laiwu pigs

120 d vs 240 d	Differentially methylated genes				
	Promoter	Exons	Introns	Downstream	Intergenic
Hyper-methylated	239	125	539	32	1078
Hypo-methylated	230	125	437	16	851

**Table 7:** Top 15 Gene Ontology (GO) categories enriched for genes with DMRs in their promoters

Category	Term	*p*-value	Genes
BP^a^	negative regulation of transcription from RNA polymerase II promoter	2.72E-04	*TNF*, *TRIM29*, *ANKRD2*, *CBX4*, *SMAD3*, *NFIX*, *CXXC5*, *HIC1*, *LEP*, *HDAC4*, *POU5F1*, *HOXA7*, *RIPPLY3*, *SEMA4D*, *BCOR*, *ZFHX3*, *SIM2*
BP	negative regulation of myotube differentiation	9.00E-03	*HDAC4*, *PLPP7*, *ANKRD2*
BP	negative regulation of osteoblast differentiation	9.70E-03	*HDAC4*, *TNF*, *SMAD3*, *SEMA4D*
BP	lipid metabolic process	1.21E-02	*FASN*, *LEP*, *LCN12*, *GDPD3*, *FADS6*
CC^b^	anchored component of plasma membrane	1.21E-02	*HYAL2*, *NTNG1*, *NTNG2*
BP	negative regulation of cell proliferation	1.26E-02	*HDAC4*, *CDKN2B*, *SMARCB1*, *IRF6*, *COPS8*, *RIPPLY3*, *FOXO4*, *CBFA2T3*, *SIRT2*
BP	negative regulation of cell adhesion	1.41E-02	*DACT2*, *PLXNB3*, *SEMA4D*
BP	positive regulation of osteoclast differentiation	1.41E-02	*TNF*, *GNAS*, *PPARGC1B*
BP	ossification	1.64E-02	*ALOX15*, *MGP*, *EXT2*, *PPARGC1B*
BP	regulation of cell shape	1.67E-02	*WASF3*, *BAIAP2*, *GNA12*, *PLXNB3*, *SEMA4D*, *ARAP1*
MF^c^	transcription corepressor activity	1.70E-02	*HDAC4*, *SKOR1*, *POU5F1*, *BCOR*, *CBFA2T3*, *DAXX*
BP	embryonic pattern specification	2.02E-02	*SMAD3*, *RIPPLY3*, *SIM2*
MF	sequence-specific DNA binding	2.85E-02	*HDAC4*, *FOXI2*, *LOC100513063*, *POU5F1*, *HOXA7*, *CXXC5*, *MAFK*, *FOXO4*, *ZFHX3*, *PITX2*
BP	immune response	3.21E-02	*FYB*, *CSF3*, *CCR7*, *IL2RA*, *SMAD3*, *TGFBR3*, *VAV1*, *CCL17*
MF	RNA polymerase II transcription factor activity	3.30E-02	*FOXI2*, *LOC100512558*, *NFIX*, *FOXO4*, *NHLH1*, *SIM2*, *PITX2*

^a^BP: biological process; ^b^CC: cellular component; ^c^MF: molecular function

### Integration analysis of transcriptome and DNA methylome in porcine LD muscle between 120 d and 240 d

**Fig. 8:**
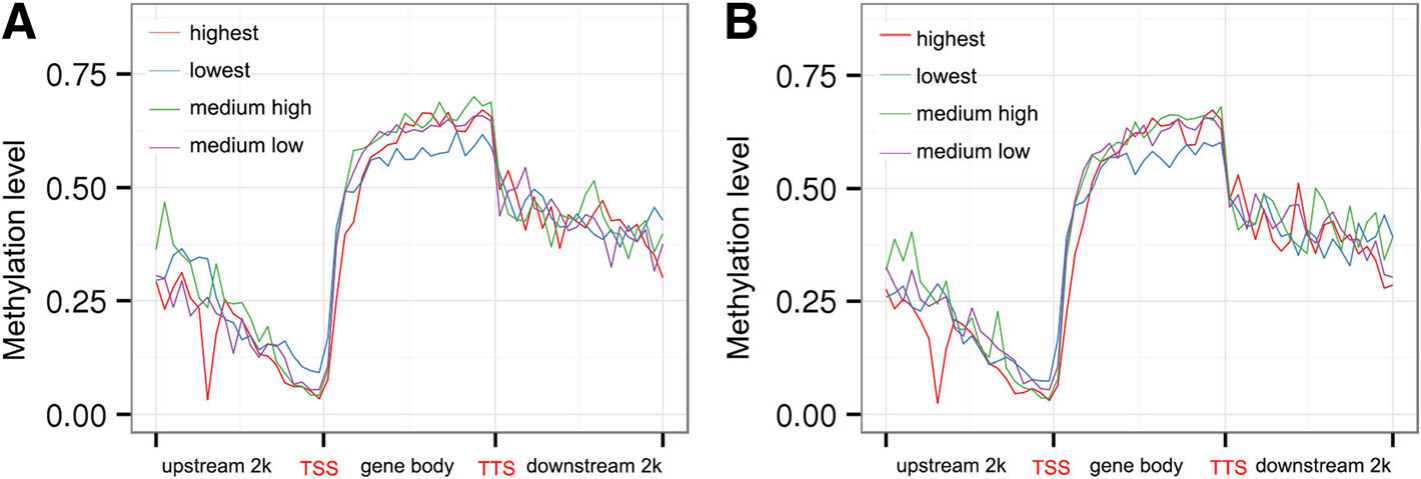
CpG methylation of genes with different expression level between 120 d and 240 d. Gene expression level was divided into four equal parts according to FPKM value. The red, blue, green, purple line indicate the highest, lowest, medium high and medium low FPKM value, respectively. **a** 120 days old Laiwu pigs; and (**b**) 240 days old Laiwu pigs. TSS: transcription start sites; TTS: transcription termination site

The gene expression level was divided into four groups according to FPKM (highest, lowest, medium high, medium low) to analyze the relationship between methylation variation and mRNA expression levels of genes in LD muscle (Additional file 14: Table S10). The expression level of the genes among the four groups was significantly different. Analysis showed that the lowest methylation level was found around the TSS in both 120 d and 240 d groups, the highest FPKM group was significantly different from others (Additional file 15: Figure S5), with an obviously sharp decrease in their upstream regions (Fig. 8). Furthermore, we calculated the proportions of DEG with DMR (4.3%), DEG without DMR (22.4%) and DMR without DEG (73.3%) (Additional file 16: Figure S6), and detected a total of 127 DEGs with DMRs, suggesting a likely role of methylation on transcription (Additional file 17: Table S11). The lipid biosynthesis relevant genes showed different DNA methylation level in gene body or intergenic region on 120 d and 240 d, such as *FASN*, *PVALB*, *ID2*, *SH3PXD2B* and *EGR1* (Table 8).

**Table 8:** The fat relevant genes with DMRs located in between 120 d and 240 d

Gene symbol	Log_2_(FC)^a^	*p*-value (DEG)	DMR width	Methylation difference	*p*-value (DMR)	Region
*FASN*	1.79	5.00E-05	9	−0.37	8.39E-10	Promoter (2-3 kb)
*PVALB*	2.45	5.00E-05	18	0.50	2.15E-07	Promoter (2-3 kb)
*LSS*	1.03	5.50E-04	15	−0.35	5.04E-13	Promoter (1-2 kb)
*ACSF3*	0.90	1.85E-03	82	−0.34	8.39E-08	Intron
*GPER1*	1.54	3.00E-04	26	−0.34	1.86E-06	3′ UTR
*IGFBP7*	0.93	4.90E-03	44	0.33	3.56E-06	Intron
*ID2*	1.27	1.50E-04	108	−0.39	9.78E-13	Distal Intergenic
*SH3PXD2B*	1.64	5.00E-05	67	−0.46	1.41E-15	Distal Intergenic
*EGR1*	3.25	5.00E-05	142	−0.40	2.85E-45	Intergenic
*PEMT*	1.09	5.00E-05	85	−0.31	4.50E-12	Distal Intergenic
*ITGB2*	1.22	1.00E-04	99	−0.34	2.32E-10	Distal Intergenic

^a^FC: Fold-Change of FPKM in transcriptome data in 120 d vs 240 d

### RRBS data validation of *EGR1* and *FASN* via BSP

**Fig. 9:**
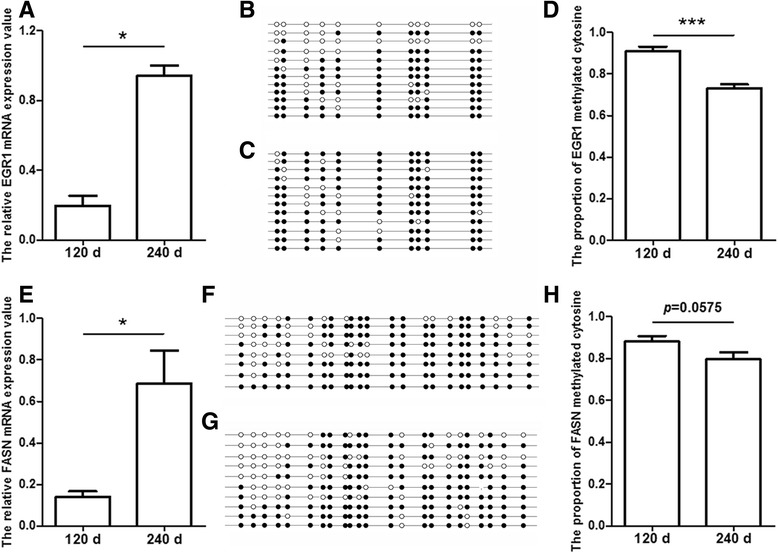
The detection of mRNA expression and methylation of candidate genes. **a** The relative mRNA expression of *EGR1* between 120 d and 240 d Laiwu pigs; The (**b**)120 d and (**c**) 240 d *EGR1* intergenic CpG methylation patterns; (**d**) The methylated cytosine proportion of *EGR1* intergenic CpG region between 120 d and 240 d. **e** The relative mRNA expression of *FASN* between 120 d and 240 d Laiwu pigs; The (**f**)120 d and (**g**) 240 d *FASN* promoter CpG methylation patterns; (**h**) The methylated cytosine proportion of *FASN* promoter CpG region between 120 d and 240 d. *p < 0.05; ***p < 0.001

According to transcriptome and RRBS data, the two candidate IMF genes *EGR1* and *FASN* were differentially expressed and with DMRs in 120 d vs 240 d. Herein, they were validated through qRT-PCR and BSP. The mRNA expression level of *EGR1*and *FASN* were up-regulated in 240 d (Fig. 9a, e), in consistent with transcriptome results. BSP analysis revealed that the proportion of CpG methylation in the intergenic region of *EGR1* was significantly lower in the LD muscle of 240 d pigs than that of 120 d (*p* < 0.0001), and that of *FASN* promoter region was not significantly different (*p* = 0.0575, Fig. 9d, h). The BSP analysis results on *EGR1* and *FASN* were basically consistent with RRBS data*.*

## Discussion

In this study, by a combinational use of transcriptome and DNA methylome analysis, we screened the genes and pathways related to IMF deposition in the LD muscle from Laiwu pigs across four developmental satges. The Laiwu pigs exhibit strong capacity to deposit fat in skeletal muscles, therefore, are ideal animal model for analyzing mechanisms underlying IMF deposition. To identify genes that are related to IMF deposition in Laiwu pigs, we first obtained transcriptome of LD muscle from Laiwu pigs of 60, 120, 240 and 400 days old, and selected genes with similar dynamics to IMF; then compared the DEGs between adjacent stages, especially focused on the genes and pathways between 120 d and 240 d, the stage of quickly increased IMF deposition; and finally analyzed the DNA methylation changes in the IMF related genes in the LD muscle from 120 d to 240 d.

By STEM analysis on the transcriptome data of four developmental stages, we found that three temporal expression profiles correlated with IMF variation in the LD muscle. From these profiles, 15 DEGs with known function in lipid metabolism were identified. Among these genes, *ACACA*, *SCD*, *ACLY*, *ELOVL1* and *FASN* are lipogenesis genes, all of them involved in long-chain fatty-acyl-CoA biosynthetic process and played regulatory and/or catalysis roles in fatty acid biosynthesis [4, 29–31]. Four genes (*SCD*, *FADS1*, *FADS2*, and *FADS3*) are known to encode desaturases, responding for the biosynthesis of unsaturated fatty acids [32–36]. Six genes (*CYP51*, *DHCR24*, *EBP*, *HSD17B7*, *SOAT1* and *SQLE*) are involved in steroid biosynthesis pathway, execute their catalytic function during cholesterolopoiesis process [37, 38]. The expression dynamics of these genes from 60 d to 400 d are in accordance with the trend of IMF deposition dynamics in the LD muscle of Laiwu pigs. Moreover, since the growth and development of LD muscle is gradually increased from 60 d to 400 d of Laiwu pigs, reflected in the changes in skeletal muscle fibre area, genes involved in myoblast fusion and muscle tissue development were also identified (Additional file 3: Figure S2), which will be further investigated in future study.

IMF deposition in skeletal muscle results from balance among the uptake, synthesis and oxydrolysis of lipids, and the development of IMF is a complex multi-organ process regulated by coordinated actions of muscle, adipocyte, and connective tissues; angiogenesis and immune system-related genes and pathways are also involved [4, 8, 39]. For instance, animals with high muscularity generally display a reduced developmental of IMF [4], suggesting that IMF candidate genes may negatively regulate muscle development. Blood vessels growth and remodelling not only supply optimal levels nutrients and oxygen to nourish adipocytes, but also provide adipose precursor and stem cells that control adipose tissue mass and function [18, 40, 41]. Accordingly, we observed the enrichment of multi-pathways in the LD muscle of Laiwu pigs from 60 d to 400 d, including cell proliferation and immune (from 60 d to 120 d), phagocytosis and protein metabolism (from 120 d to 240 d) (Fig. 4).

The 120 d vs 240 d group has the most DEGs and mostly up-regulated in 240 d. The fat deposition pathways firstly emerged in this period, concordant with the quick increase of IMF content, representing a critical fat deposition period. The fatty acid biosynthesis relevant DEGs were identified in this group, such as *FASN*, *ACACA*, *ACLY* and *SCD*. *FASN* (fatty acid synthase) catalyzes all of the reaction steps in the synthesis of palmitate from acetyl-CoA and malonyl-CoA, which plays a central role in de novo lipogenesis in mammals [29], and was reported to be related with pork IMF content [42, 43]. *ACACA* (Acetyl-CoA carboxylase alpha) is a key regulator of de novo fatty acid synthesis [30, 44] and the haplotype *A*_*4899*_ *C*_*5196*_ can decrease IMF percentage in a Duroc commercial population [45]. *ACLY* (ATP citrate lyase) is a rate-limiting lipogenic enzyme in pigs associated with fatty acid biosynthesis [31], its effects on IMF deposition has not been studied. Notably, *SCD* (stearoyl-coenzyme A desaturase) is also a rate-limiting enzyme in lipogenesis, regulating the biosynthesis of C16: 1 and C18: 1 from C16: 0 and C18: 0, respectively, and this process is regulated by SREBF1 [33, 34]. Bessa et al. [35] reported that IMF is positively related to SCD protein expression level in commercial pig breeds. Therefore, it is reasonable to speculate that it is the relative highly expressed SCD that triggers the increase of MUFA percentage [46], particularly between 120 and 240 days old Laiwu pigs.

Besides these well-known IMF relevant genes, we also focused on dramatically up- or down-regulated genes that probably relevant to fat metabolisms, such as *LDLR*, *FOSL1*, *EGR1*, *G0S2*, *FAM213B* and *SORBS1*. *LDLR* (low density lipoprotein receptor) plays an important role in lipid transportation and shows high positive correlation with IMF content in Piau pigs [47]. *FOSL1* (FOS like 1, AP-1 transcription factor subunit) could be activated by the upstream AP1 motif, induced by Liver X receptors (LXRs) in mouse, which involves in regulating lipid synthesis and transport [48]. Boyle et al. [49] showed that *EGR1* (early growth response transcription factor 1) is a negative regulator during 3 T3-L1 differentiation [50]. *G0S2* (G0/G1 switch gene 2) can attenuate the activity of the intracellular triacylglycerol hydrolase and adipose triglyceride lipase [51]. And the role of the gene *FAM213B* (family with sequence similarity 213 member B) in fat deposition was rarely reported, which deserves further study. While the down-regulated gene *SORBS1* (sorbin and SH3 domain containing 1) plays a key role in adipogenesis [52], and the expression level in the visceral adipose depots correlated negatively with body mass index [53]. Taken together, only *LDLR* has been reported related with IMF content among the six mentioned genes, the function of the other five genes in IMF deposition still deserves further investigations.

The well studied TFs relevant to lipid metabolism, such as *KLF5*, *SREBF2*, *TP53* and *TWIST1*, were also identified. *KLF*s (Krüppel-like factors) regulate adipocyte differentiation in mammals, and KLF5 acts as a key regulator, by binding to SREBP1, to enhance the SREBP1-mediated increase in *FASN* promoter activity [54]. *SREBF*s (sterol regulatory element binding factors) are major regulators of carbohydrate and lipid metabolism [30]. *SREBF2* is closely associated with cholesterol biosynthesis [55], showing an activation Z-score of 3.51 in comparison between 120 d and 240 d. *TP53* (tumor protein p53) is involved in pig myogenesis [56] and functional p53 can regulate cellular glucose metabolism through several proteins such as TP53-induced glycolysis and phosphoglycerate mutase [57]. *TWIST1* (twist family BHLH transcription factor 1) was demonstrated as a regulator during the adipocytes gene expression in 3 T3-L1 [58, 59]. The roles of these TFs in IMF deposition of pigs have not been reported.

It has been well studied that DNA methylation plays an important role in the regulation of gene expression [21, 23]. In this study, we obtained the DNA methylation landscapes of LD muscle in 120 d and 240 d Laiwu pigs. The results showed that most of the DMRs located in intergenic regions, which is consistent with another study [18]. The methylation level of CpG dinucleotide around TSS region showed a ‘V’ shaped curve in the LD muscle of both 120 d and 240 d Laiwu pigs, this pattern was also reported in other species, such as human [60], cattle [23] and rat [61]. Moreover, the DMRs located in promoter region were mainly involved in muscle, adipose and bone development, implying that methylation probably plays an important role during the LD muscle development.

Generally, the genes with high expressed levels are often associated with a relative lower promoter methylation [23, 62], while for the gene body regions, it remains unclear [63–65]. Studies assumed that DNA methylation in gene body is positively correlated with gene expression level, because DNA methylation might alter chromatin structure and transcription elongation efficiency [66, 67]. In this study, the expressed genes showed the lowest methylation level around TSS, and for the highly mRNA expressed genes both in the LD muscles of 120 d and 240 d, the methylation level in the promoter region was decreased, which is consistent with previous reports [63, 68, 69]. Integration analysis of transcriptome and methylome revealed a set of candidate IMF deposition genes probably regulated by methylation, such as *FASN*, *ID2*, *ITGB2*, *PEMT* and *SH3PXD2B*. These mentioned genes were all hypomethylated in 240 d in the promoter or intergenic regions. *ID2* is an adipogenic transcription factor that stimulates PPAR expression and adipocyte differentiation [70], loss of ID2 expression leads to decreased white adipose tissue development and adipogenesis, both in vitro and in vivo [71]. ITGB2 was an up-regulated adipokine associated to obesity [72]. And SH3PXD2B positively regulates the fat cell differentiation [73]. The role of DNA methylation on the expression of these genes requires further investigations.

In this study, we performed transcriptome comparison on LD muscle from four developmental stages, and DNA methylome comparison between two developmental stages, three individuals in each stage. Although a relatively high correlation was observed in each group, ideal analysis should be performed on muscle biopsies collected in the same pig at all developmental stages to eliminate the individual effect. Function of the IMF related genes identified by this study will be further characterized on cell level.

## Conclusions

In summary, this study provides a comprehensive landscape of transcriptome of the LD muscle in Laiwu pigs ranging from 60 d to 400 d. We identified 127 DEGs related to lipid biosynthesis in 120 d vs 240 d, including genes *FASN*, *ACACA*, *ACLY* and *SCD*, and transcription factors *EGR1*, *SREBF2*, *TP53* and *TWIST1.* The fat biosynthesis relevant genes *FASN*, *PVALB*, *ID2*, *SH3PXD2B* and *EGR1,* showed differences in DNA methylation in the gene body or intergenic regions from 120 d to 240 d in Laiwu pigs. The role of these genes in porcine IMF deposition needs further investigations.

## Methods

### Animal sampling and meat composition measurements

A total of 12 pure castrated male Laiwu pigs reared under similar environmental and feeding conditions in Laiwu Pig Conservation Center (Laiwu, Shandong) were randomly selected at four developmental stages: 60, 120, 240 and 400 days of age (*n* = 3). Each stage includes three individuals of similar body weights. These pigs were weighed and electrically stunned to ameliorate the suffering of the animals before death, then blood drawn and sampled from the LD muscle at the third lumbar vertebra, and vacuum-packed in a low-oxygen environment until subsequent IMF content measurement by Soxhlet petroleum ether extraction after sampling 24-h [74, 75]. Similarly, four DYL pigs of 120 days of age were randomly selected from Xingyue Pig Breeding Co. Ltd. (Taian, Shandong). The DYL pigs were weighed, electrically stunned, blood drawn and slaughtered, all efforts were made to minimize suffering. Immediately after slaughter, a sample from the LD muscle at the third lumbar vertebra was collected and frozen in liquid nitrogen for further study.

Total lipids were extracted from the LD samples using a benzene-petroleum ether (1:1) mixture. The lipids were directly methylated using 0.4 mol/L KOH methyl alcohol solution according to Demirel et al. [76]. Fatty acid methyl esters (FAME) were quantified using a gas chromatograph (GC) equipped with a DB-17 column from Agilent Technologies (30 m × 0.25 mm × 0.25 μm; Palo Alto, CA, USA) on a Shimadzu GC-2010 (Kyoto, Japan). The fatty acid composition was analyzed with an Automatic Amino Acid Analyzer, and the operating steps were performed as previously described [77].

### Total RNA extraction and RNA-sequencing

Total RNA was extracted from Laiwu pig muscle samples (stored at −80 °C) according to the EZNA Tissue RNA Kit instruction manual (Omega-Biotech, Doraville, USA). RNA concentration and purity were measured with a NanoDrop 2000 Spectrophotometer (Thermo Fisher Scientific, Wilmington, DE). RNA integrity was assessed using the RNA Nano 6000 Assay Kit with the Agilent Bioanalyzer 2100 system (Agilent Technologies, CA, USA).

RNA sequencing libraries were generated using the NEBNext Ultra RNA Library Prep Kit for Illumina (New England Biolabs, Ipswich, MA, USA) with multiplexing primers, according to the manufacturer’s instructions. Sequencing was performed using a paired-end 125-cycle rapid run on the Illumina HiSeq2500 (Illumina Inc., San Diego, CA, USA). Low quality reads were removed by perl script, and the clean reads were filtered from the raw reads and mapped to the *Sus scrofa* genome (Sscrofa10.2) using Tophat2 software [78]. Gene expression levels were estimated based on the FPKM values obtained using Cufflinks software [79]. Here, only genes with an absolute value of log_2_ (Fold-Change) ≥ 2 and an FDR < 0.01 were used for subsequent analysis. The transcriptome data have been deposited with the NCBI Gene Expression Omnibus (GEO, http://www.ncbi.nlm.nih.gov/geo) under accession number GSE90135. Additionally, the transcriptome data of IB × DU pigs at 120 d were downloaded from GEO of NCBI (GSE86441) to compared with the Laiwu pig at the same age [16]. The DEGs were also screened based on log2FC ≥ 2 and an FDR < 0.01.

### DNA preparation and RRBS sequencing

The genomic DNAs were extracted using the TIANamp genomic DNA kit (Tiangen, Beijing, China) following the manufacturer’s instructions of the 120 and 240 days old pig muscle tissues, based on the phenotypic and transcriptome data showed that this stage was the IMF deposition fastest period. The construction of DNA methylation library was performed at Biomarker (Beijing, China) using RRBS. The RRBS library was constructed using 2 μg of high-quality genomic DNA, pooling with an equal amount from three 120 and 240 days old pigs, respectively. Briefly, DNA was restriction digested using MspI enzyme, which cut the DNA at sites CCGG, then the fragment were end-repaired and dA-tailing to blunt-end products, followed by adaptor-ligation with T overhang. The ligation products were purified by 2% agarose gel electrophoresis and size-selected of DNA fragments 150–400 bp long (including 100 bp adaptor). Size-selected DNA was bisulfite-conversion with the NEXTflex Bisulfite-Seq Kit (Bioo Scientific, Austin, TX, USA). The final library was generated by PCR-amplified, enriching for fragments with adapters on both ends and the RRBS was performed by Illumina HiSeq X Ten (Illumina Inc., San Diego, CA, USA). The clean reads were aligned to the pig reference genome (Sscrofa10.2) (ftp://ftp.ensembl.org/pub/release-75/fasta/sus_scrofa) and produced by the BS-seeker2 v.2.0.8 using Bowtie2 v.2.1.0 [80] in local alignment mode and no more than 2 mismatches per read. Methylation status was determined using weighted methylation level [81]. The DMRs were produced, which sites coverage depth more than 10×, and at least 3 different methylation sites and Fisher’s exact test *p* < 0.05 using MOABS [27]. The RRBS sequences were submitted to Gene Expression Omnibus (GEO) of NCBI under the study accession number GSE93563.

### Bioinformatics analysis

The DEGs in 60 d vs 120 d, 120 d vs 240 d, and 240 d vs 400 d comparisons were uploaded to DAVID Bioinformatics Resources (version 6.8, https://david.ncifcrf.gov/home.jsp) to pathway enrichment analysis. STEM software (version 1.3.9, http://www.cs.cmu.edu/~jernst/stem) was used to classify all of the genes into profiles with similar expression patterns [24], and the correlation coefficients of significant clustered profiles were calculated using the MultiExperiment Viewer (version 4.9.0, http://www.tm4.org). All of the DEGs were analyzed using Cytoscape ClueGO plug-in (version 2.3.2; http://apps.cytoscape.org/apps/cluego) as a complementary analysis method. Only Benjamini-corrected values of *p* < 0.05 were considered statistically significant. The obtained IMF candidate genes were compared with pig IMF relevant QTL dataset to validate fat metabolism genes. Finally, the upstream regulators prediction was predicted by Ingenuity Pathway Analysis (Ingenuity Systems, Redwood City, CA, USA; http://www.ingenuity.com), a bioinformatics tool for biological functions, canonical pathways and gene networks. Meanwhile, the TFs binding sites were predicted using JASPAR (http://jaspar.genereg.net). The DMRs were blasted [82] and pathway enriched also using online DAVID analysis.

### qRT-PCR

Quantitative PCR was performed to validate the DEGs according to the Pearson’s correlation between the FPKM from the RNA-Seq data and the relative expression data obtained using qRT-PCR. First-strand cDNA was synthesized using Primescript RT reagent (Takara Bio Inc., Otsu, Japan) in a 20 μl total volume, containing 1 μL total RNA, 1 μL gDNA Eraser, 2 μL 5 × gDNA Eraser Buffer, 4 μL 5 × Prime Script Buffer 2, 1 μL Prime Script RT Enzyme Mix, 1 μL RT Primer Mix4 and RNase-Free dH_2_O. The expression of six genes was quantified using SYBR Premix Ex Taq (Takara Bio Inc., Otsu, Japan) in Agilent Mx3000P system (Agilent Co., Wilmington, Delaware, USA) in a total volume of 25 μL containing 12.5 μL 2× Pre Ex Taq, 0.5 μL ROX II, 2 μL cDNA, 0.5 μL each of the forward and reverse primer (10 μM) and dH_2_O. The standard curve for each gene was used to confirm amplification specificity and efficiency, and an appropriate 3-fold dilution ratio was used. The data were normalized to GAPDH and RPL7, and the experiments were run in triplicate. The 2^-∆∆CT^ method was used to calculate relative gene expression. Concordance correlation coefficient [83] was calculated between FPKM and qRT-PCR experiments.

### Validation of RRBS data using BSP

We performed BSP to validate the RRBS results. The methylation primers were designed using Methyl Primer Express v1.0, which were provided in Additional file 18: Table S12. The bisulfite conversion of pig genome DNA was executed according to protocol of EZ DNA Methylation-Gold Kit (Zymo Research, Irvine, CA, USA). Then amplification converted DNA using Ex Taq Hot Start Version (Takara Bio Inc., Otsu, Japan). And the PCR product was cloned into the pMD18-T vector (Takara Bio Inc., Otsu, Japan). Twenty subclones were selected for each fragment and the positive clones were sequenced using ABI3730XL DNA Analyzer (ABI, CA, USA). All the sequences were analyzed using BiQ Analyzer v2.0.

### Statistical analysis

The live weight, IMF content and fatty acid composition over four developmental stages were performed with one-way ANOVA, followed by Duncan’s multiple range test (*p* < 0.05) by using SAS 8.2 (SAS Institute, Cary, NC, USA). Student *t*-test was employed to analyze the mRNA over four developmental stages of pig muscle. The GO and KEGG analysis were performed by Fisher’s *t*-test. *p* < 0.05 was considered significantly different.

## Additional files

Additional file 1: Figure S1. Validation of the expression of candidate genes using qRT-PCR. The Y-axis on the left side of the histogram represents the gene expression level according to qRT-PCR (marked as *), and the right Y-axis of line represents the standard value of FPKM based on transcription (indicated as #). The Pearson’s correlation coefficient (r) and Gene Symbol are shown above the figure. #/*p < 0.05; ##/**p < 0.01; ###/***p < 0.001. (PNG 2842 kb)

Additional file 2: Table S1. The four significantly clustered profiles and the corresponding genes across four developmental stages in Laiwu pigs. A total of 10 profiles were generated and only four profiles significantly clustered across 60, 120, 240 and 400 days old in Laiwu pigs. (XLSX 76 kb)

Additional file 3: Figure S2. The differentially expressed genes and functions associated with muscle metabolism in profile 1, 3 and 4. GO terms and genes are represented as nodes based on their kappa score more than 0.4 and networks with at least three nodes. The node size represents the GO terms enrichment significance. (PNG 1770 kb)

Additional file 4: Table S2. The differentially expressed genes (DEGs) in 60 d vs 120 d, 120 d vs 240 d and 240 d vs 400d. We showed all DEGs only with absolute value of log2 (Fold-Change) ≥ 1 and FDR ≤ 0.01 in adjacent development stages of Laiwu pigs. (XLSX 81 kb)

Additional file 5: Table S3. The fat relevant differentially expressed genes (DEGs) with similar expression trend in the comparison 120 d vs 240 d of Laiwu pigs. (XLSX 12 kb)

Additional file 6: Table S4. The upstream regulators and their target genes in 120 d vs 240 d Laiwu pigs. (XLSX 11 kb)

Additional file 7: Table S5. The TFs binding sites predicted by JASPAR database. (DOCX 280 kb)

Additional file 8: Figure S3. The IMF content between DYL and Laiwu pig breeds. *p < 0.05. (PNG 5 kb)

Additional file 9: Table S6. All the DEGs between IB × DU and Laiwu pig breeds. The DEGs were screened with absolute value of log2 (Fold-Change) ≥ 1 and FDR ≤ 0.01. (XLSX 281 kb)

Additional file 10: Table S7. The IMF candidate genes between IB × DU and Laiwu pig breeds. (DOCX 17 kb)

Additional file 11: Figure S4. Distribution of CpG methylation in the LD muscle of 120 d (a) and 240 d (b) Laiwu pigs across pig chromosomes. (PNG 1168 kb)

Additional file 12: Table S8. The differentially methylated cytosines (DMCs) and corresponding genes in 120 and 240 days old Laiwu pigs. The DMCs were produced by sequencing depth ≥ 4×, and FDR ≤ 0.05. (XLSX 6864 kb)

Additional file 13: Table S9. The differentially methylated regions (DMRs) in 120 and 240 days old Laiwu pigs. The DMRs were generated by sequencing depth ≥ 10×, at least 3 DMCs, meanwhile the minimum value ≥ 0.3, and *p* < 0.05 using Fisher’s exact test. (XLSX 461 kb)

Additional file 14: Table S10. The classification of transcriptome according to their FPKM value at 120 d and 240 d of Laiwu pigs. (XLSX 2057 kb)

Additional file 15: Figure S5. The ANOVA test among the highest, medium high, medium low and lowest FPKM groups in 120 d vs 240 d Laiwu pigs. ***p < 0.001. (PNG 10 kb)

Additional file 16: Figure S6. The proportion of DEGs with DMRs, DEGs without DMRs and DMRs without DEGs in 120 d vs 240 d Laiwu pigs. (JPEG 75 kb)

Additional file 17: Table S11. Description of DEGs with DMRs, DEGs without DMRs and DMRs without DEGs in 120 d vs 240 d Laiwu pigs. (XLSX 1094 kb)

Additional file 18: Table S12. The primers for methylation analysis on intergenic region of EGR1 and promoter region of FASN by BSP. (DOCX 14 kb)

## Abbreviations

BP: Biological process
CC: Cellular component
DEGs: Differentially expressed genes
DMCs: Differentially methylated cytosines
DMRs: Differentially methylated regions
DYL: Duroc × Yorshire × Landrace
FC: Fold-change
FDR: False discovery rate
FPKM: Fragments Per Kilobase of transcript per Million mapped reads
GO: Gene ontology
IMF: Intramuscular fat
IPA: Ingenuity Pathway Analysis
KEGG: Kyoto encyclopedia of genes and genomes
LD: *longissimus dorsi*
MF: molecular function
MUFA: Monounsaturated fatty acids
PUFA: Polyunsaturated fatty acids
RRBS: Reduced representation bisulfite sequencing
SFA: Saturated fatty acids
STEM: Short Time-series Expression Miner
TFs: Transcription factors
TSS: Transcription start sites
TTS: Transcription termination site
