# Supplementary figures and images for: Dynamic transcriptome and DNA methylome analyses on longissimus dorsi to identify genes underlying intramuscular fat content in pigs
Source: BMC Genomics. 2017 Oct 12;18:780. doi: 10.1186/s12864-017-4201-9 (PMC5639760; doi:10.1186/s12864-017-4201-9)

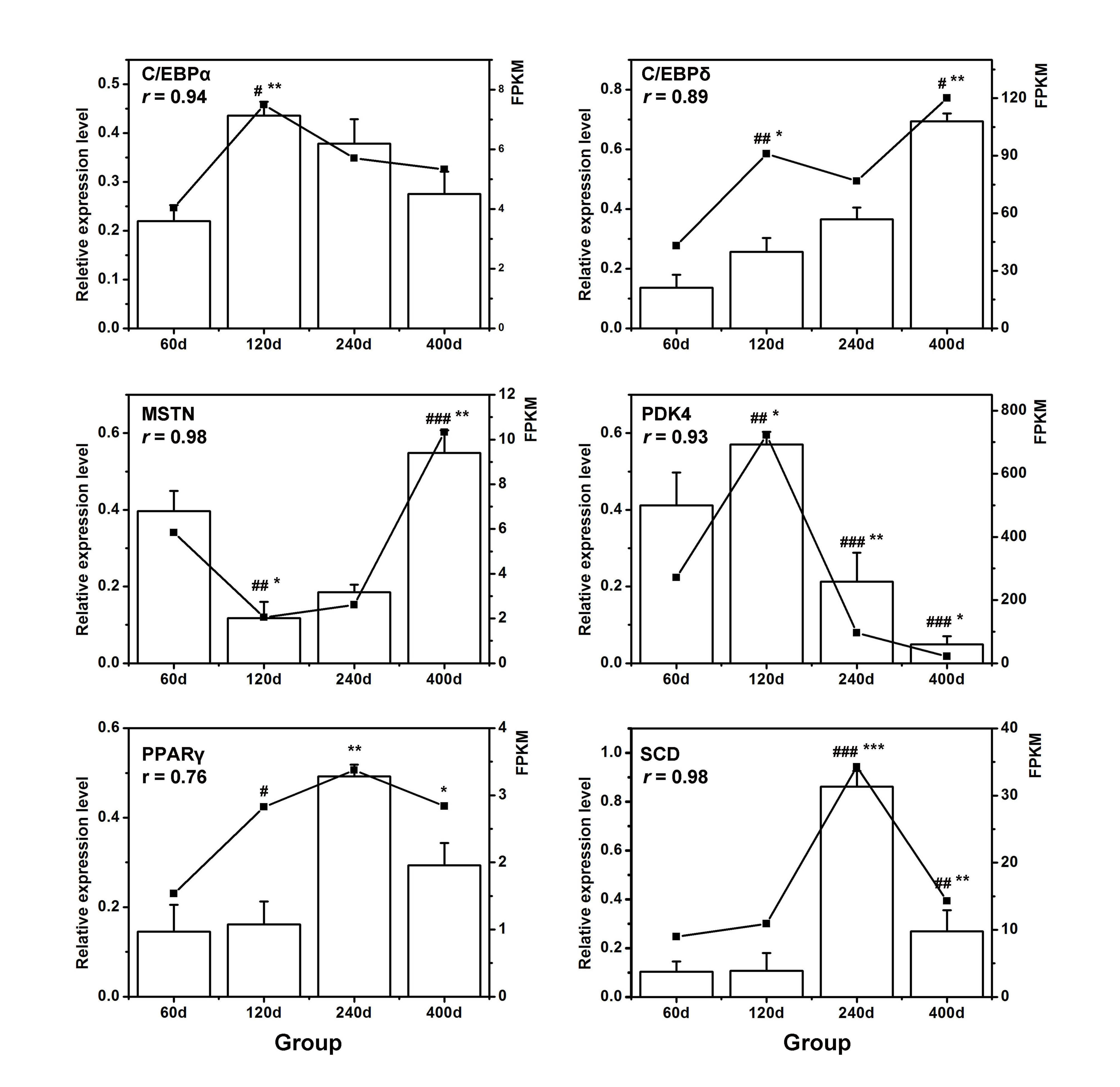

Supplement: Supplementary file 1 — Validation of the expression of candidate genes using qRT-PCR. The Y-axis on the left side of the histogram represents the gene expression level according to qRT-PCR (marked as *), and the right Y-axis of line represents the standard value of FPKM based on transcription (indicated as #). The Pearson’s correlation coefficient (r) and Gene Symbol are shown above the figure. #/*p < 0.05; ##/**p < 0.01; ###/***p < 0.001. (PNG 2842 kb) [file 12864_2017_4201_MOESM1_ESM.png]

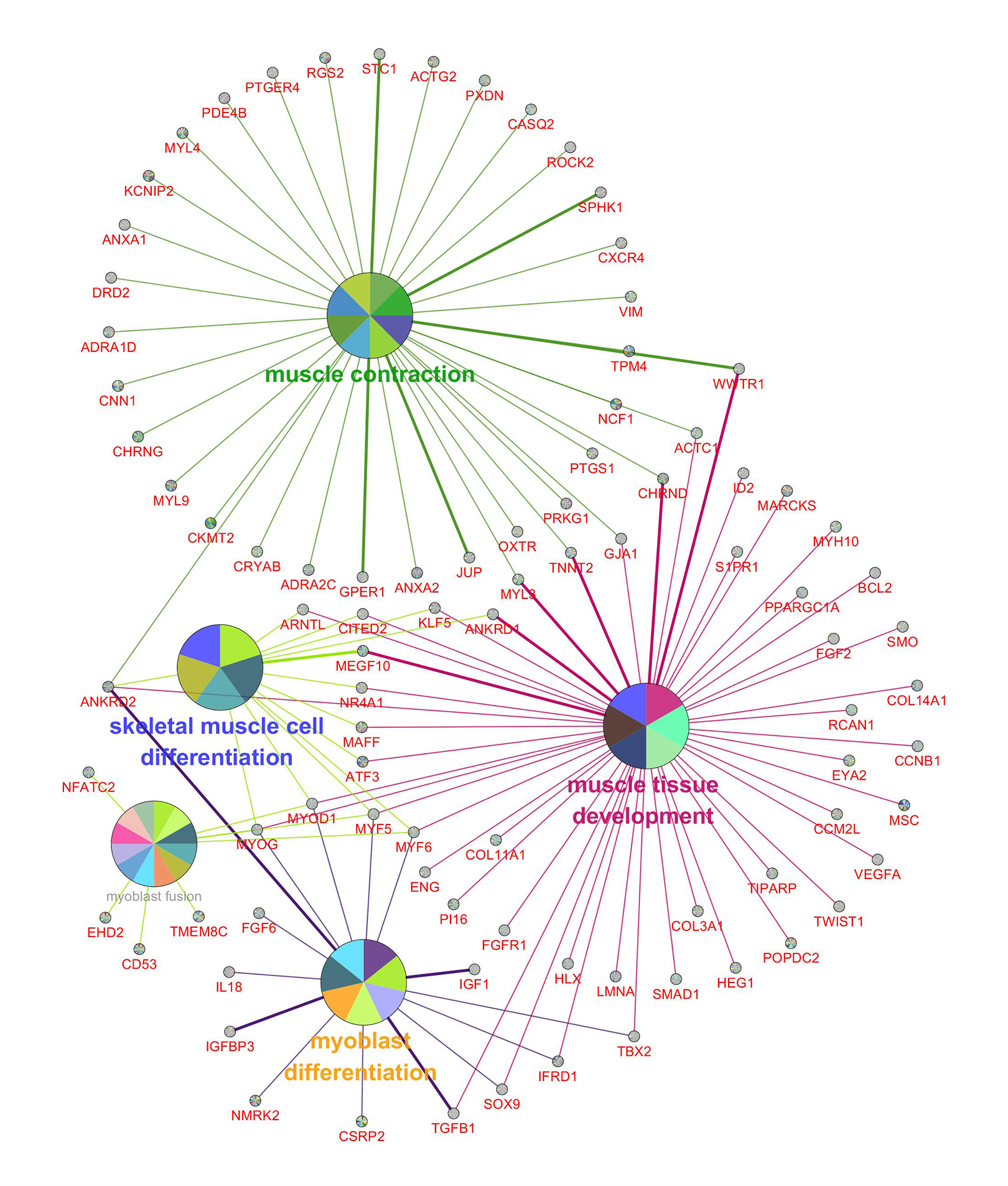

Supplement: Supplementary file 3 — The differentially expressed genes and functions associated with muscle metabolism in profile 1, 3 and 4. GO terms and genes are represented as nodes based on their kappa score more than 0.4 and networks with at least three nodes. The node size represents the GO terms enrichment significance. (PNG 1770 kb) [file 12864_2017_4201_MOESM3_ESM.png]

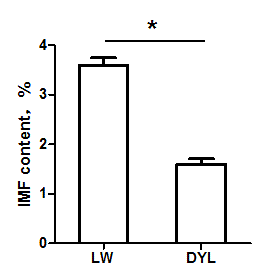

Supplement: Supplementary file 8 — The IMF content between DYL and Laiwu pig breeds. *p < 0.05. (PNG 5 kb) [file 12864_2017_4201_MOESM8_ESM.png]

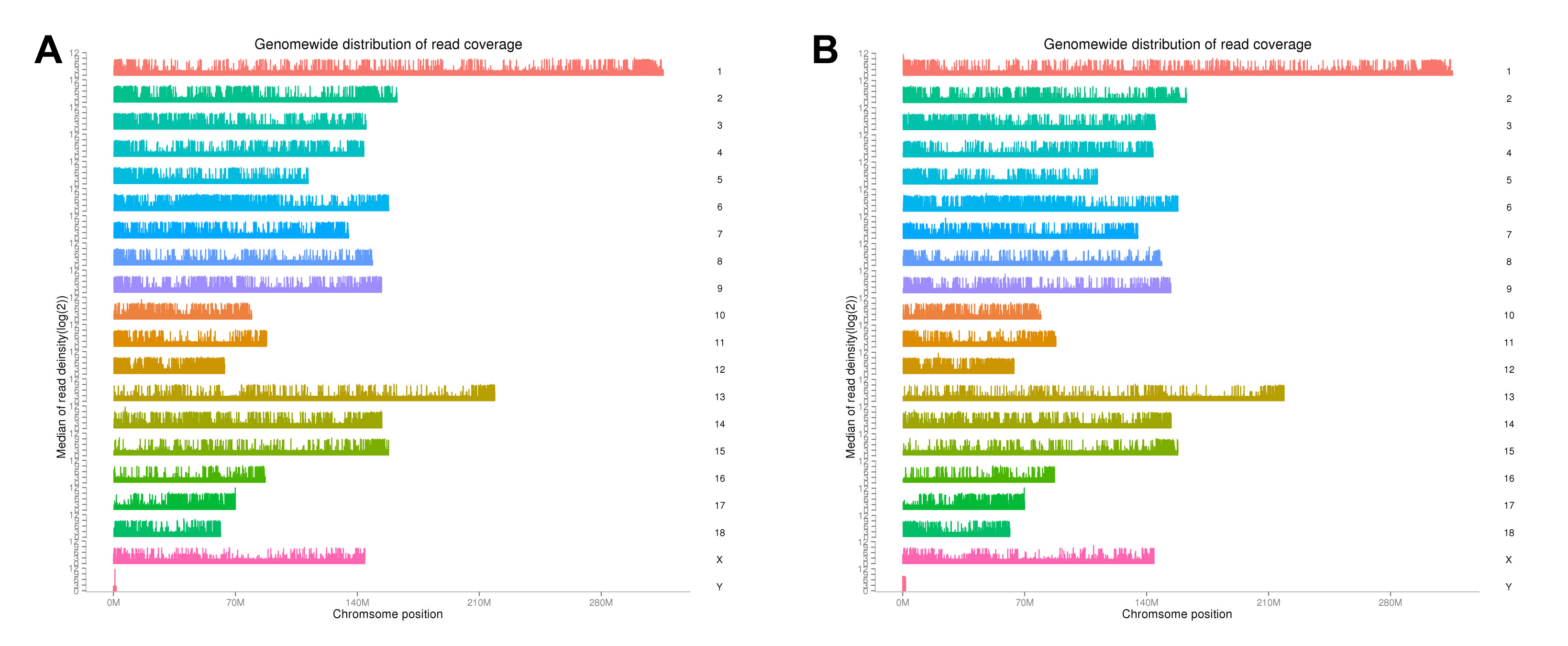

Supplement: Supplementary file 11 — Distribution of CpG methylation in the LD muscle of 120 d (a) and 240 d (b) Laiwu pigs across pig chromosomes. (PNG 1168 kb) [file 12864_2017_4201_MOESM11_ESM.png]

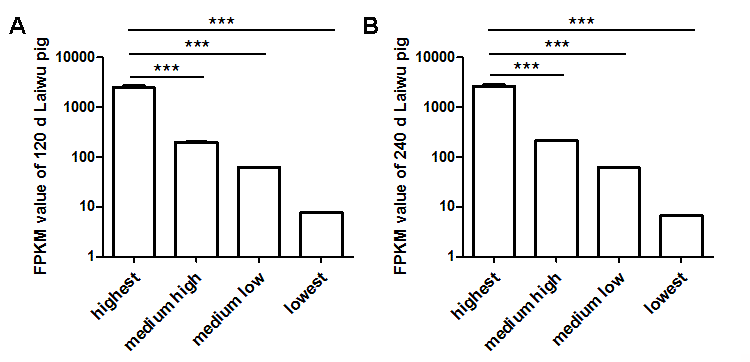

Supplement: Supplementary file 15 — The ANOVA test among the highest, medium high, medium low and lowest FPKM groups in 120 d vs 240 d Laiwu pigs. ***p < 0.001. (PNG 10 kb) [file 12864_2017_4201_MOESM15_ESM.png]

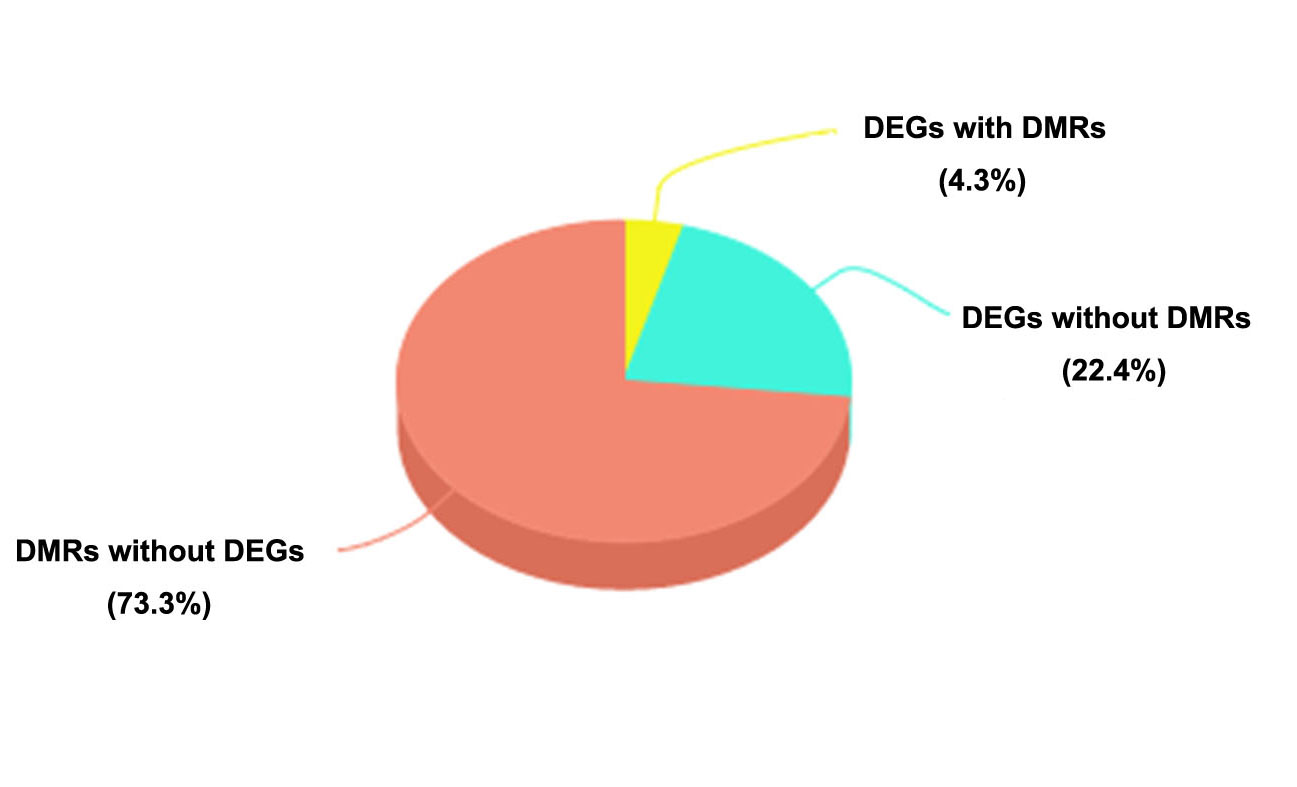

Supplement: Supplementary file 16 — The proportion of DEGs with DMRs, DEGs without DMRs and DMRs without DEGs in 120 d vs 240 d Laiwu pigs. (JPEG 75 kb) [file 12864_2017_4201_MOESM16_ESM.jpg]
