# Supplementary material for: Dynamic transcriptome and DNA methylome analyses on longissimus dorsi to identify genes underlying intramuscular fat content in pigs
Source: BMC Genomics. 2017 Oct 12;18:780. doi: 10.1186/s12864-017-4201-9 (PMC5639760; doi:10.1186/s12864-017-4201-9)
Supplement: Supplementary file 7 — The TFs binding sites predicted by JASPAR database. (DOCX 280 kb) [file 12864_2017_4201_MOESM7_ESM.docx]

Table S5. The TFs binding sites predicted by JASPAR database.

| ID | Name | Species^1^ | Class | Sequence logo |
| --- | --- | --- | --- | --- |
| MA0162.1 | Egr1 | 10090 | Zinc-coordinating | 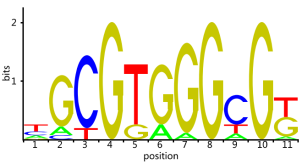 |
| MA0162.2 | EGR1 | 9606 | C2H2 zinc finger factors | 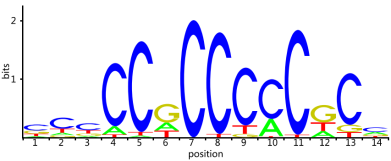 |
| PB0010.1 | Egr1-1 | 10090 | Zinc-coordinating | 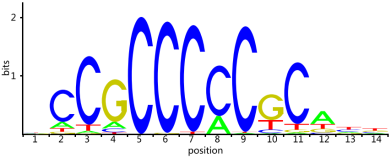 |
| PB0114.1 | Egr1-2 | 10090 | Zinc-coordinating | 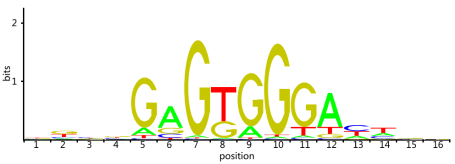 |
| MA0617.1 | Id2 | 10090 | Basic helix-loop-helix factors (bHLH) | 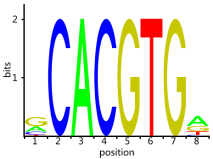 |
| MA0599.1 | KLF5 | 9606 | C2H2 zinc finger factors | 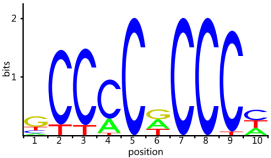 |
| MA0664.1 | MLXIPL | 9606 | Basic helix-loop-helix factors (bHLH) | 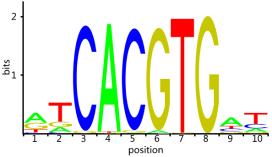 |
| MA0596.1 | SREBF2 | 9606 | Basic helix-loop-helix factors (bHLH) | 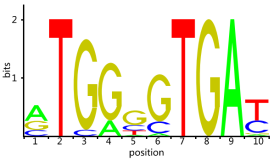 |
| MA0828.1 | SREBF2 | 9606 | Basic helix-loop-helix factors (bHLH) | 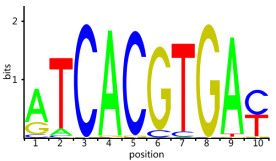 |
| MA0595.1 | SREBF1 | 9606 | Basic helix-loop-helix factors (bHLH) | 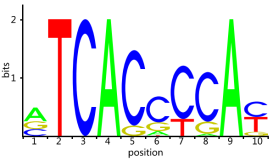 |
| MA0829.1 | Srebf1 | 10090 | Basic helix-loop-helix factors (bHLH) | 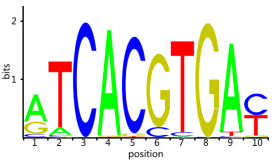 |
| MA0106.1 | TP53 | 9606 | Zinc-coordinating | 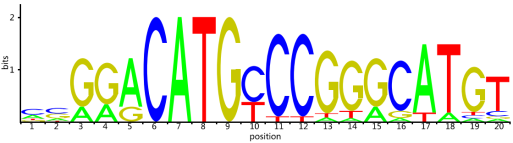 |
| MA0106.2 | TP53 | 9606 | Zinc-coordinating | 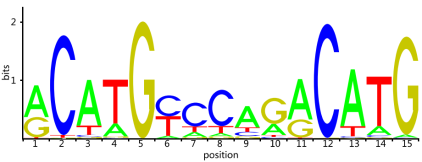 |
| MA0106.3 | TP53 | 9606 | p53 domain factors | 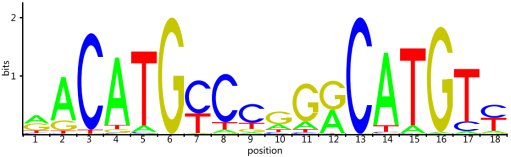 |

^1^Species: The species based on predicted transcription factor binding sites. The 10090 and 9606 represent *Mus musculus* and *Homo sapiens*, respectively.
