# Supplementary material for: Dynamic transcriptome and DNA methylome analyses on longissimus dorsi to identify genes underlying intramuscular fat content in pigs
Source: BMC Genomics. 2017 Oct 12;18:780. doi: 10.1186/s12864-017-4201-9 (PMC5639760; doi:10.1186/s12864-017-4201-9)
Supplement: Supplementary file 10 — The IMF candidate genes between IB × DU and Laiwu pig breeds. (DOCX 17 kb) [file 12864_2017_4201_MOESM10_ESM.docx]

Table S7. The IMF candidate genes between IB×DU and Laiwu pig breeds

| Gene | FDR | log_2_FC^1^ | Function |
| --- | --- | --- | --- |
| *UCP3* | 4.32E-03 | -1.3539 | fatty acid metabolic process (GO:0006631) |
| *PRKCA* | 8.78E-13 | -2.5645 | positive regulation of lipid catabolic process (GO:0050996) |
| *PRKAG3* | 7.45E-03 | -1.2678 | fatty acid biosynthetic process (GO:0006633) |
| *PPARGC1A* | 7.66E-03 | -1.9814 | adipocytokine signaling pathway ko04920 |
| *PLCXD3* | 6.88E-04 | -1.6952 | lipid metabolic process (GO:0006629) |
| *PEX19* | 3.69E-04 | -1.3869 | negative regulation of lipid binding (GO:1900131) |
| *ME1* | 9.37E-03 | -1.1326 | PPAR signaling pathway (ko03320) |
| *ILVBL* | 6.91E-04 | -1.6510 | fatty acid alpha-oxidation (GO:0001561) |
| *FOXO1* | 4.43E-03 | -1.0924 | negative regulation of fat cell differentiation (GO:0045599) |
| *DBI* | 2.82E-03 | -1.1365 | lipid transport (GO:0006869) |
| *CRYZ* | 1.20E-03 | -1.2149 | fatty acid metabolic process (GO:0006631) |
| *TCF4* | 1.33E-08 | 1.7316 | negative regulation of fat cell differentiation (GO:0045599) |
| *SOAT1* | 6.96E-03 | 1.2910 | fatty-acyl-CoA binding (GO:0000062) |
| *SNAI2* | 2.17E-05 | 2.5566 | white fat cell differentiation (GO:0050872) |
| *SLC2A1* | 2.08E-03 | 1.5945 | adipocytokine signaling pathway (ko04920) |
| *SIK2* | 1.28E-30 | 3.5645 | fatty acid homeostasis (GO:0055089) |
| *SH3GLB2* | 3.58E-03 | 1.0217 | phospholipid biosynthetic process (GO:0008654) |
| *RGS2* | 4.92E-12 | 2.4342 | brown fat cell differentiation (GO:0050873) |
| *PSKH1* | 1.34E-04 | 1.4874 | lipid biosynthetic process (GO:0008610) |
| *PPT1* | 7.51E-03 | 1.4300 | lipid catabolic process (GO:0016042) |
| *PLK2* | 2.36E-03 | 1.8214 | lipid biosynthetic process (GO:0008610) |
| *PIM1* | 8.68E-03 | 2.1821 | lipid biosynthetic process (GO:0008610) |
| *PDIK1L* | 9.67E-03 | 1.0142 | lipid biosynthetic process (GO:0008610) |
| *NR4A2* | 1.89E-12 | 3.1247 | cellular response to lipid (GO:0071396) |
| *NR2F1* | 4.26E-03 | 1.0044 | response to lipid (GO:0033993) |
| *NR1D1* | 2.05E-03 | 1.0822 | long-chain fatty acid transport (GO:0015909) |
| *LPCAT1* | 7.20E-03 | 1.1014 | lipid biosynthetic process (GO:0008610) |
| *KLF9* | 4.18E-10 | 1.8807 | fat cell differentiation (GO:0045444) |
| *ITGAV* | 1.05E-06 | 1.7366 | glycerophospholipid metabolic process (GO:0006650) |
| *ITGA3* | 1.29E-03 | 1.3435 | glycerolipid metabolic process (GO:0046486) |
| *IRS1* | 5.75E-05 | 1.3157 | adipocytokine signaling pathway ko04920 |
| *ID4* | 7.53E-03 | 1.2291 | negative regulation of fat cell differentiation (GO:0045599) |
| *HES1* | 2.79E-04 | 1.6219 | regulation of fat cell differentiation (GO:0045598) |
| *FN1* | 8.63E-03 | 1.2433 | fatty acid metabolic process (GO:0006631) |
| *FAR2* | 1.54E-04 | 1.3879 | long-chain fatty-acyl-CoA metabolic process (GO:0035336) |
| *FAM213B* | 4.72E-05 | 1.3807 | Arachidonic acid metabolism (ko00590) |
| *FADS3* | 2.67E-03 | 1.1574 | fatty acid biosynthetic process (GO:0006633) |
| *EGF* | 4.82E-04 | 1.1592 | lipid metabolic process (GO:0006629) |
| *DCLK2* | 1.01E-03 | 1.1819 | lipid biosynthetic process (GO:0008610) |
| *CYB5R3* | 5.21E-03 | 1.0151 | unsaturated fatty acid biosynthetic process (GO:0006636) |
| *CREM* | 2.12E-08 | 1.7299 | positive regulation of lipid biosynthetic process (GO:0046889) |
| *CEBPB* | 7.44E-28 | 3.3689 | brown fat cell differentiation (GO:0050873) |
| *CCND1* | 2.57E-04 | 1.8612 | fat cell differentiation (GO:0045444) |
| *C3* | 4.40E-10 | 1.9879 | positive regulation of lipid storage (GO:0010884) |
| *C1QTNF9* | 1.20E-03 | 1.1254 | fat cell differentiation (GO:0045444) |
| *B4GALNT2* | 5.50E-03 | 2.1515 | lipid glycosylation (GO:0030259) |
| *ATP8A1* | 4.08E-08 | 2.1726 | phospholipid transport (GO:0015914) |
| *ATF7* | 5.87E-03 | 1.0055 | fat cell differentiation (GO:0045444) |

^1^log_2_FC: The logarithms of FPKM fold-change between IB×DU and Laiwu pigs at 120 d and the control group is IB×DU.
