# Supplementary material for: Dynamic transcriptome and DNA methylome analyses on longissimus dorsi to identify genes underlying intramuscular fat content in pigs
Source: BMC Genomics. 2017 Oct 12;18:780. doi: 10.1186/s12864-017-4201-9 (PMC5639760; doi:10.1186/s12864-017-4201-9)
Supplement: Supplementary file 18 — The primers for methylation analysis on intergenic region of EGR1 and promoter region of FASN by BSP. (DOCX 14 kb) [file 12864_2017_4201_MOESM18_ESM.docx]

Table S12 The primers for methylation analysis on intergenic region of *EGR1* and promoter region of *FASN* by BSP.

| Gene | Primer | Products |
| --- | --- | --- |
| FASN-F1-meth | AGGTAGAGGGTTTGTAGTTAGGG | 409bp |
| FASN-R1-meth | TTTCAAAAAAAACAAAAACCTCA |  |
| FASN-F2-meth | AGGGTTTGTAGTTAGGGTAGGA | 320bp |
| FASN-R2-meth | AAATAACCTCCAACAACATCC |  |
| EGR1-F1-meth | TGTTTGGTTTTTTTATATTTTTTTT | 308bp |
| EGR1-R1-meth | AAACTAAACAAACTACAACTCTCC |  |
| EGR1-F2-meth | TTTTTTTAAGATTTTTTTTGGGTAGG | 260bp |
| EGR1-R2-meth | AAAAAACAAACCCTTTTTCCATC |  |
